# Assessing scale and predictive diversity in models for single-cell transcriptomics based on Geneformer

**DOI:** 10.1371/journal.pcbi.1013701

**Published:** 2026-07-30

**Authors:** Junfan Chen, Fabian Schmidt, Ricardo Henao

**Affiliations:** 1 Biomedical Sciences Division (BioMed), King Abdullah University of Science and Technology (KAUST), Thuwal, Saudi Arabia; 2 Department of Biostatistics & Bioinformatics, Duke University, Durham, North Carolina, United States of America; University of Western Ontario: Western University, CANADA

## Abstract

Single-cell transcriptomic data provide critical insights into cellular states and disease mechanisms, and foundation models have recently emerged as powerful tools for learning gene–gene relationships from these data. However, current approaches often overlook key challenges, including the mismatch between model design and the rank-ordered structure of gene expression profiles, as well as the unclear benefits of large-scale pretraining for biological applications. Here, we present GF_CAB_, a modified modeling framework designed to better capture the structural properties of ranked single-cell transcriptomic data. GF_CAB_ incorporates a cumulative assignment mechanism to suppress repeated gene predictions and a similarity-based regularization strategy to promote diversity in model outputs. Across multiple evaluation settings, including pretraining behavior, biologically relevant classification tasks, and cross-dataset analyzes, GF_CAB_ consistently reduces redundancy and enhances the recovery of low-frequency genes with known functional and disease relevance while maintaining or improving predictive accuracy. In downstream applications, including classification and zero-shot batch effect correction, the model achieves competitive or improved performance compared to existing approaches. We further show that indiscriminately increasing the pretraining data scale does not uniformly improve performance. Instead, models trained on substantially smaller datasets can match or exceed the performance of larger models and often demonstrate improved generalization across datasets. Together, these findings highlight the importance of aligning model design with the intrinsic structure of biological data and suggest that architectural innovation can reduce reliance on large-scale training data. GF_CAB_ provides a framework for developing more efficient and biologically informative models for single-cell analysis, with potential applications in disease characterization and precision biology.

## Author summary

Single-cell analysis helps researchers understand how genes work together inside individual cells, and recent artificial intelligence models have shown strong potential for uncovering these patterns. However, many existing approaches do not fully account for how this data is structured, and often assume that using more training data will always improve performance. In this study, we introduce GF_CAB_, a model designed to better match the way single-cell data are organized. By reducing repeated predictions and encouraging the model to consider a wider range of genes, GF_CAB_ produces more diverse and biologically meaningful results, including the identification of less common but important gene signals. We also show that simply increasing the amount of training data does not always lead to better outcomes. Instead, well-designed models trained on smaller datasets can perform just as well and often generalize better to new data. These findings highlight the importance of model design in developing efficient and reliable tools for single-cell research.

## Introduction

Characterizing genetic relationships within single-cell transcriptomic data [1,2], and leveraging these representations for a broad spectrum of downstream biological tasks, has emerged as a central paradigm across modern computational biology and related disciplines [3–5]. To this end, a wide range of computational approaches has been developed to systematically learn latent genetic structures from such data and generalize these representations to predictive tasks, including perturbation response modeling, drug sensitivity prediction, disease classification, and cellular dynamics inference [6–9]. Early efforts adapted classical frameworks, such as multilayer perceptrons (MLPs) and differential expression testing (DET) [10], while subsequent work introduced specialized models tailored to single-cell data, including scVI, scANVI, and GEARS [11–13], collectively demonstrating the feasibility of extracting biological representations from sparse transcriptomic profiles. More recently, single-cell transcriptomic foundation models have been proposed as a unified representational framework, leveraging self-attention mechanisms [14] to capture complex gene–gene dependencies and enabling flexible adaptation to a wide range of downstream tasks through pretraining and fine-tuning. Emerging models such as scGPT, scFoundation, and SCmilarity [4,15,16] highlight the potential of this paradigm. In parallel, the Bidirectional Encoder Representations from Transformers (BERT) architecture [17] has been adapted to single-cell transcriptomics. An early example, scBERT [18], applied a BERT-style framework to single-cell data and demonstrated its utility for cell-type annotation, while the subsequent Geneformer (GF), a ranking-based BERT variant pretrained on approximately 30 million cells [19], extended this paradigm toward foundation-model applications across a broader range of downstream tasks.

Despite these advances, foundation models such as GF remain susceptible to fundamental mismatches between backbone design choices and the intrinsic ranking properties of single-cell transcriptomic data, an issue that has yet to be systematically examined. In particular, GF operates on rank-ordered gene expression profiles, yet directly inherits the masked language modeling (MLM) objective from natural language processing [20], where token repetition is permissible. This misalignment introduces undesirable behaviors: GF can repeatedly predict the same gene within a single cellular profile, despite the biological constraint that each gene is uniquely represented per cell. Consequently, the model exhibits a bias toward highly frequent and ubiquitously expressed genes, leading to reduced prediction diversity and diminished biological interpretability. In addition, recent studies in foundation models have highlighted that indiscriminate scaling of pretraining data does not necessarily yield improved generalization performance [21–23]. Within the context of single-cell transcriptomic modeling, this issue remains largely unexplored. Current approaches predominantly emphasize increasing dataset size, often aggregating ever-larger collections of cells, yet lack a principled assessment of whether such scaling is necessary or effective for representation learning in this domain.

**Fig 1:**
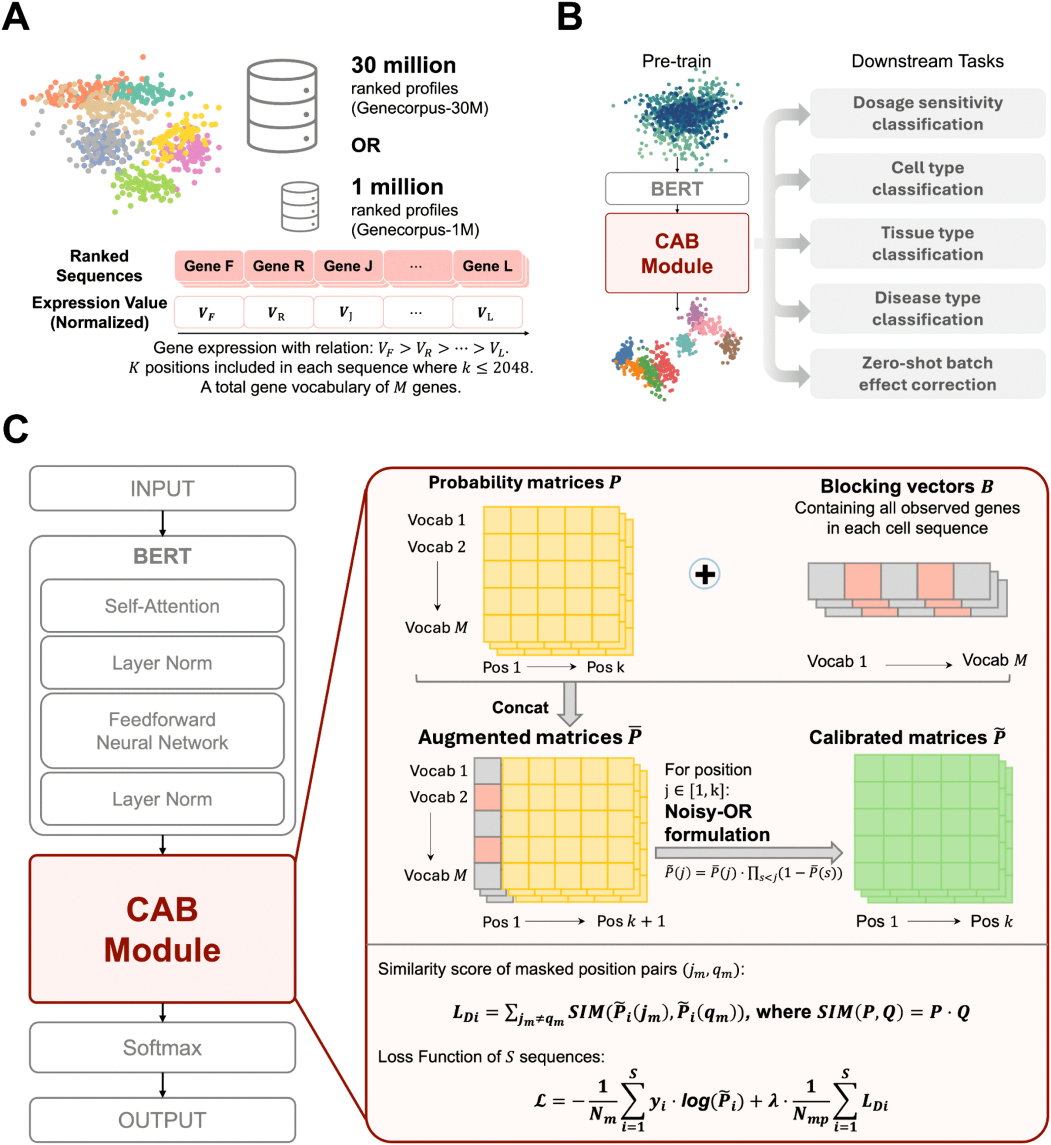
Modeling framework and evaluation pipeline overview. **(A)** Pretraining is performed on two data scales, 1 million (1M) and 30 million (30M) single-cell profiles, where each cell is represented as a rank-ordered gene sequence based on expression levels, with a vocabulary exceeding 20,000 genes. **(B)** Pretrained models are evaluated across multiple downstream tasks, including gene dosage sensitivity classification, cell type classification, tissue type classification, disease type classification, and zero-shot batch integration. **(C)** Illustration of the GF_CAB_ architecture, highlighting the cumulative-assignment suppression and similarity-based regularization components introduced during pretraining to reduce repetition and enhance predictive diversity. All icons and graphical elements in this figure were created by the authors or generated using Python scripts.

To address these underexplored limitations, we introduce GF_CAB_, a modified GF architecture designed to better align model behavior with the rank-ordered structure of single-cell transcriptomic data. Central to this design is a cumulative assignment and balancing (CAB) module, implemented as a post-prediction processor. The CAB module incorporates a probability cumulative-assignment mechanism to propagate positional constraints across predictions, ensuring consistency with the non-redundant nature of gene rankings within each cell. In parallel, a similarity-based regularization term penalizes redundant or overly similar probability distributions across gene positions, thereby promoting diversity in predicted gene identities. Together, these components explicitly encode structural priors of the data into the prediction process, mitigating the mismatch introduced by conventional masked language modeling objectives. To further investigate the role of data scale in conjunction with architectural design, we additionally construct a reduced pretraining corpus (Genecorpus-1M) by uniformly subsampling one million profiles from the original Genecorpus-30M, enabling controlled comparisons across different data regimes (Fig 1A).

We show that integrating the CAB module effectively alleviates the aforementioned limitations, as evidenced by systematic improvements in masked gene prediction behavior, including reduced repetition and enhanced diversity. To evaluate downstream utility, we benchmark GF_CAB_ across a range of biologically relevant tasks, including gene dosage sensitivity prediction, cell type classification, tissue type classification, disease classification, and zero-shot batch effect correction (Fig 1B). Although ranking-based models remain inherently constrained in zero-shot generalization settings, GF_CAB_ consistently matches or outperforms the original GF across tasks. Furthermore, we examine the role of pretraining data scale by comparing models trained under varying dataset sizes. Our results indicate that increased data scale does not uniformly translate to performance gains. In contrast, targeted architectural modifications, such as the CAB module, can substantially narrow and, in some cases, eliminate performance gaps associated with data scale, achieving competitive or superior performance even under up to 30-fold reductions in pretraining data while maintaining stronger generalization capacity. Collectively, these findings establish GF_CAB_ as a principled and data-aligned extension of GF, demonstrating that incorporating structural constraints into model design can serve as an effective alternative to indiscriminate data scaling in single-cell transcriptomic foundation models.

## Results

### GF_CAB_ is a model architecture aligned with rank-ordered single-cell transcriptomic profiles

GF_CAB_ is designed to address two key limitations of the original Geneformer (GF) architecture: repetitive gene predictions across masked positions within a single ranked cell profile and a systematic bias toward highly frequent, ubiquitously expressed genes at the expense of rarer but biologically informative signals. These issues arise from the mismatch between the masked language modeling objective and the non-redundant, rank-ordered structure of single-cell transcriptomic data. To mitigate this mismatch, GF_CAB_ operates on the post-BERT probability distributions and introduces a cumulative assignment and balancing (CAB) module to enforce cross-position constraints and promote distributional diversity (Fig 1C; Methods).

Formally, for a cell profile with multiple masked positions, the BERT backbone produces a probability matrix over the gene vocabulary for each position, where each column represents a predicted distribution across all candidate genes. To reduce repetition across masked positions, we introduce a cumulative-assignment mechanism that explicitly propagates information across positions. Specifically, a blocking vector encoding observed (unmasked) genes is incorporated to suppress candidates that are already present within the same cell profile. The resulting probability distributions are then sequentially adjusted so that genes assigned high confidence at earlier positions are progressively down-weighted in subsequent predictions. This cumulative suppression, conceptually analogous to a Noisy-OR formulation [24], ensures that high-probability assignments are not repeatedly selected across positions, thereby enforcing non-redundant predictions within each cell.

In parallel, to enhance predictive diversity, we incorporate a similarity-based regularization term that penalizes overly similar probability distributions across masked positions. Concretely, the similarity between positional predictions is quantified using pairwise dot-product similarity, and the aggregated similarity across positions is minimized during training. This constraint discourages the model from concentrating probability mass on a limited subset of highly frequent genes. Instead, it encourages a broader exploration of candidate genes across positions. The final training objective combines the standard masked language modeling loss with this diversity regularization, balancing predictive accuracy and distributional diversity.

Together, these two components reshape the prediction landscape by explicitly encoding structural priors of rank-ordered transcriptomic data, enabling GF_CAB_ to generate more diverse and biologically informative gene predictions while maintaining consistency with the non-redundant nature of gene expression profiles.

### GF_CAB_ improves masked gene prediction fidelity and reveals diminishing returns from data scaling

**Fig 2:**
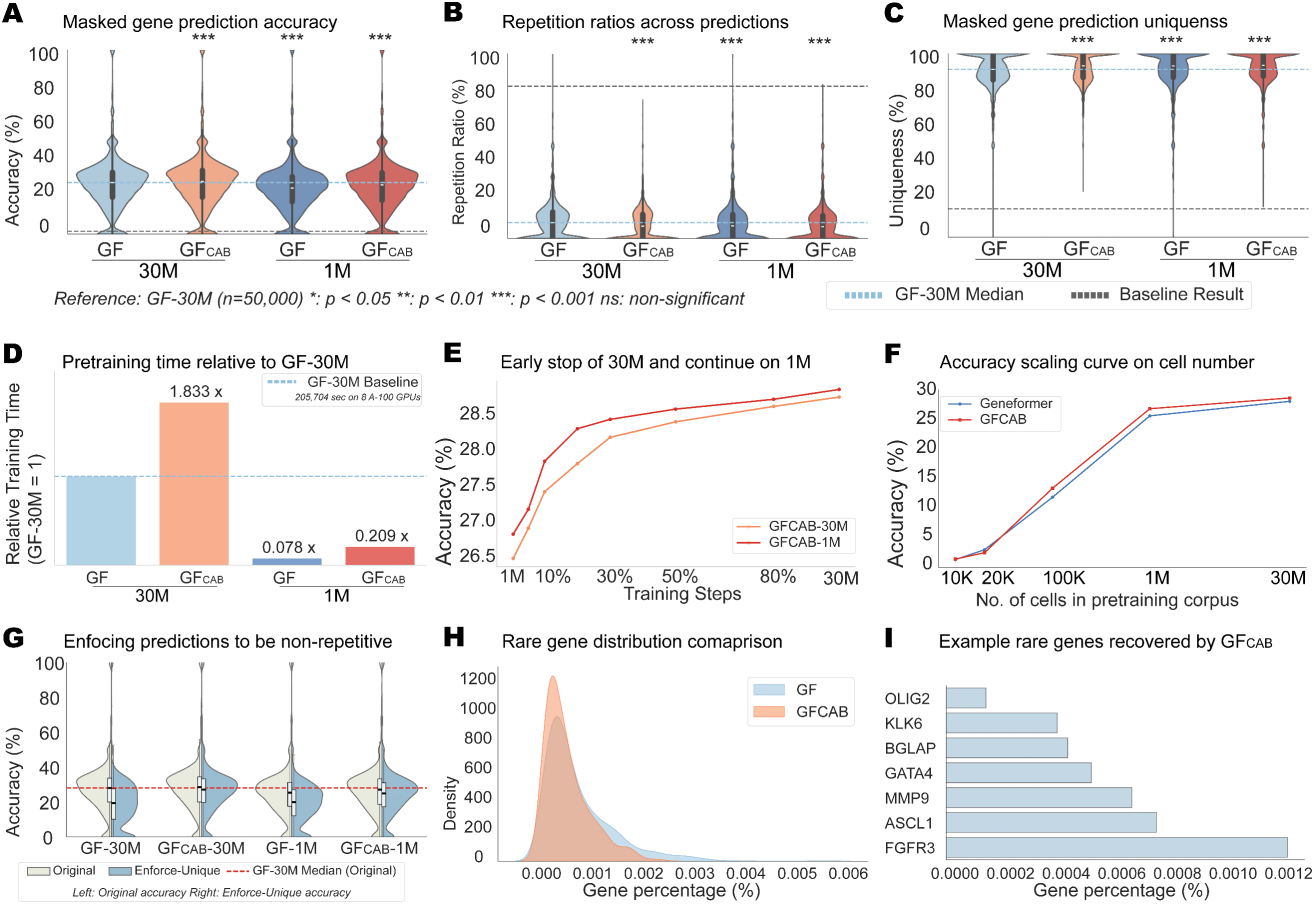
GF_CAB_ improves masked gene prediction behavior across accuracy, repetition, and diversity. **(A)** Distributions of masked token prediction accuracy for GF and GF_CAB_ at 30M and 1M pretraining scales. Wilcoxon tests were performed relative to GF-30M; white lines indicate medians and baseline accuracy. GF_CAB_ achieves consistently higher accuracy. **(B)** Distributions of repetition ratios across models and data scales, showing reduced redundancy in GF_CAB_. **(C)** Distributions of uniqueness scores, demonstrating increased predictive diversity in GF_CAB_. **(D)** Relative training time of GF and GF_CAB_, normalized to GF trained on 30M cells (GF-30M). **(E)** Effect of training dynamics: comparison between continued training on 1M data and early stopping on 30M data. Performance gains are concentrated in early training stages, with diminishing returns as training progresses. **(F)** Scaling behavior of masked token prediction accuracy with increasing pretraining data size, showing diminishing marginal gains at larger scales above 1M cells. **(G)** Comparison between standard predictions and predictions under an enforced-uniqueness constraint. While enforcing uniqueness eliminates repetition, it leads to decreased accuracy, highlighting the trade-off addressed by GF_CAB_. **(H)** Frequency distribution of low-prevalence genes predicted by GF and GF_CAB_, showing that GF_CAB_ preferentially captures rarer genes. **(I)** Representative biologically relevant genes uniquely recovered by GF_CAB_, including lineage-defining transcription factors (ASCL1, OLIG2, KLK6), tissue-specific markers (BGLAP, GATA4), and disease-associated regulators (FGFR3, MMP9).

We first evaluated whether GF_CAB_ improves alignment with rank-ordered single-cell transcriptomic profiles. To systematically characterize predictive behavior, we quantified masked gene prediction accuracy, repetition ratio, and uniqueness (Methods), capturing model fidelity, redundancy across masked positions, and the diversity of predicted genes, respectively. We benchmarked both GF and GF_CAB_, pretrained on 1M and 30M profiles, on an independent test set of 50,000 ranked cells (Fig 2A–2C, S1 Table). GF_CAB_ consistently achieved higher prediction accuracy, reduced repetition, and increased uniqueness across both data scales. These results indicate that improved architectural alignment with the rank-ordered structure directly translates into enhanced predictive performance and more biologically consistent outputs.

Notably, models trained on smaller datasets exhibited systematically higher diversity, characterized by reduced repetition and increased coverage of distinct genes (Fig 2B, 2C). This observation suggests that increasing pretraining data alone does not resolve the tendency of rank-based models to overproduce frequent genes and may instead reinforce such biases. One plausible explanation is that large-scale pretraining encourages the model to learn highly consistent and dominant gene–gene relationships, leading to sharper and more concentrated probability distributions over a limited set of high-frequency genes. In contrast, models trained on smaller datasets are exposed to fewer and less constrained co-expression patterns, resulting in smoother predictive distributions with higher entropy. This, in turn, promotes greater exploratory diversity during masked prediction, allowing lower-frequency genes to be more readily recovered. Such behavior is consistent with our empirical observations of increased uniqueness and reduced repetition in smaller-scale models. To contextualize these findings, we additionally report relative training times (Fig 2D), highlighting the trade-off between computational cost and marginal performance gains.

We next investigated the relationship between data scale and predictive performance. Tracking masked gene prediction accuracy across training steps revealed that performance gains are largely concentrated in the early phase of training, with most improvements achieved within the first ~30% of total steps, followed by clear diminishing returns (Fig 2E, S1 Fig). Across both architectures, models trained on 1M profiles consistently matched or outperformed their 30M counterparts, indicating that increased data scale does not necessarily yield improved predictive capacity under fixed model capacity. Extending this analysis across a broader range of dataset sizes (10K–30M cells) further confirmed a saturating scaling behavior, with performance gains plateauing beyond the ~1M regime (Fig 2F, S2 Fig). To further assess architectural scaling, we fixed the pretraining dataset at 30M profiles and systematically increased model capacity (S3 Fig). Performance improvements were primarily observed in lower-capacity, underparameterized regimes, suggesting that gains diminish once the model approaches saturation relative to the data scale. These results highlight the importance of coordinated scaling between model capacity and data size, rather than independent expansion of either factor. Notably, GF_CAB_ consistently outperformed baseline models across all data and model scales, underscoring the importance of architectural alignment over brute-force scaling. However, we acknowledge the risk that our subsampling strategy may benefit our smaller datasets in their diversity representation compared to curating datasets simply from a more limited number of sources.

To disentangle diversity improvements from potential metric artifacts, we compared naive predictions with those obtained under enforced uniqueness, where repeated predictions are explicitly prevented. Under this constraint, GF exhibited a substantial drop in predictive accuracy, whereas GF_CAB_ retained performance with only minor degradation (Fig 2G, S4 Fig). This result indicates that GF_CAB_ does not achieve improved diversity through post hoc redistribution alone, but rather through intrinsically better-structured predictive distributions.

Finally, we assessed the biological relevance of enhanced diversity by examining predictions across gene frequency strata. GF_CAB_ showed increased recovery of low-prevalence genes compared to GF (Fig 2H, 2I, S5 Fig), including lineage-associated transcription factors (ASCL1, OLIG2, KLK6) [25–27], tissue-specific markers (BGLAP, GATA4) [28,29], and disease-relevant signaling and remodeling mediators (FGFR3, MMP9) [30,31]. These findings suggest that improved diversity translates into greater sensitivity to biologically meaningful but underrepresented signals.

Collectively, these results demonstrate that GF_CAB_ enhances masked gene prediction fidelity by reducing redundancy and increasing diversity, while revealing that performance gains from either data scaling or model scaling are inherently limited. Instead, principled architectural alignment with data structure provides a more effective route to improving model behavior in single-cell transcriptomic foundation models.

### GF_CAB_ improves downstream task performance and preserves generalization under reduced data scaling

**Fig 3:**
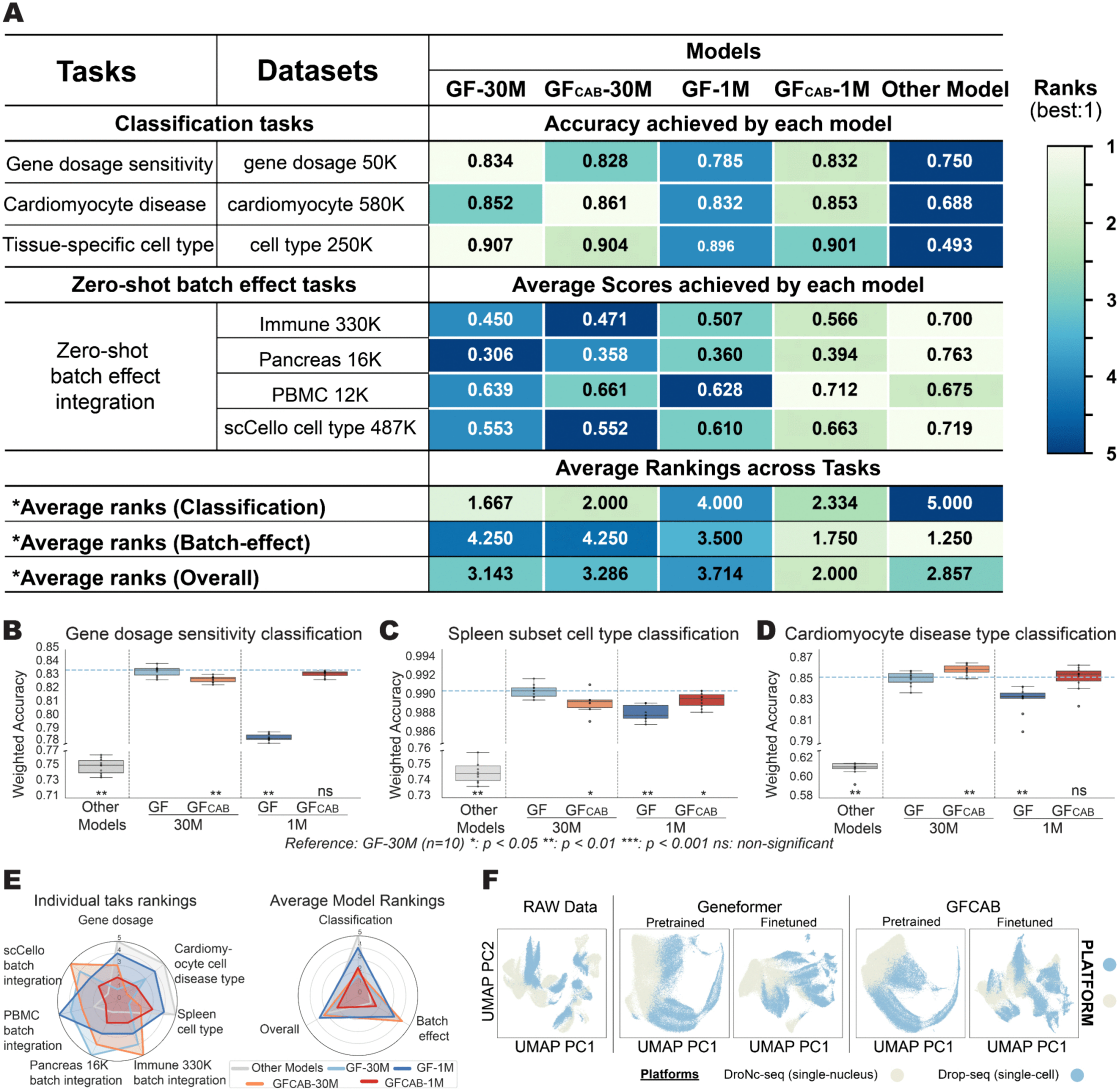
Overview of model performance across downstream tasks, model architectures, and pretraining data scales. **(A)** Summary of model performance across three classification tasks (gene dosage sensitivity, cardiomyocyte disease-state classification, and spleen cell type classification) and four zero-shot batch integration benchmarks, with numerical metrics displayed alongside color-coded ranking indicators. **(B–C)** Detailed classification results for each task, showing comparisons between foundation models and the best-performing classical baselines (SVM, Random Forest, Logistic Regression). **(E)** Per-task ranking and average ranking across all tasks and datasets, with a radar plot illustrating comparative performance across models and data scales. **(F)** Visualization of embedding quality on cardiomyocyte datasets, including both Drop-seq (single-cell) and DroNc-seq (single-nucleus) modalities. Models are fine-tuned on single-cell data and evaluated in a zero-shot manner across modalities, demonstrating improved batch integration performance after fine-tuning.

To evaluate whether improvements in pretraining behavior translate into downstream utility, we assessed GF_CAB_ across a diverse set of biologically relevant classification tasks and zero-shot batch effect correction benchmarks. Specifically, we considered three classification tasks, including gene dosage sensitivity prediction, cardiomyocyte disease-state classification, and tissue-specific cell type classification, and four zero-shot batch integration datasets, including Immune 330K [32], Pancreas 16K [33], PBMC 12K [12,34], and scCello out-of-distribution (OOD) cell type 487K [35] (Fig 3A). We benchmarked GF_CAB_ against the original Geneformer (GF) across both 1M and 30M pretraining regimes, alongside representative classical baselines including logistic regression [36], random forest [37], and support vector machine [38], as well as established reference methods including HVGs-based representations and scVI [12] for batch integration.

Across classification tasks, GF_CAB_ demonstrated competitive or improved predictive performance relative to GF (Fig 3B–3D, S2–S5 Table). For in-distribution tasks derived directly from the pretraining corpus, including gene dosage sensitivity and spleen cell type classification, GF_CAB_ showed slightly reduced performance compared to GF at the 30M scale but consistently outperformed GF under the 1M setting. This behavior can be attributed to the regularization introduced by the CAB module, which suppresses redundant predictions and reduces overly concentrated probability distributions across masked positions. While these mechanisms can improve predictive diversity and robustness, they may also reduce the extent to which the model exploits task-specific predictive signals in settings where the original GF model already performs strongly. Thus, the slight advantage of GF-30M in these in-distribution tasks may reflect differences in the trade-off between predictive diversity and task-specific optimization.

In contrast, for the out-of-distribution cardiomyocyte disease classification task, where we predict disease states (non-failing, hypertrophic, and dilated) from independently processed datasets, GF_CAB_ outperformed GF across both pretraining scales (Fig 3D). This reversal highlights the effect of regularization in mitigating overfitting to pretraining-specific patterns and improving generalization under distribution shift. These observations are consistent with a classical bias–variance trade-off, whereby increased regularization reduces overfitting at the expense of peak in-distribution performance while enhancing robustness to unseen data. Notably, GF_CAB_ pretrained on 1M data consistently matched or exceeded the performance of GF pretrained on 30M data, indicating that architectural improvements can offset, and in some cases surpass, gains from large-scale pretraining.

We next evaluated zero-shot batch effect correction as a proxy for model generalization, following established evaluation protocols [39]. GF_CAB_ consistently improved batch mixing performance relative to GF across datasets and scales (Fig 3A, S6 Table). Although ranking-based foundation models remain less competitive than dedicated integration methods [35,39,40], this improvement indicates that enhanced predictive structure translates into better cross-dataset generalization. Notably, models pretrained on 1M data exhibited stronger generalization than their 30M counterparts, suggesting that excessive data scaling may overfit dominant dataset-specific patterns and reduce transferability.

To provide a unified assessment, we aggregated performance across all tasks using a ranking-based summary (Fig 3A, 3E). GF_CAB_ pretrained on 1M data achieved the highest overall ranking when jointly considering classification accuracy and batch integration performance. This trend remained consistent when prioritizing biologically relevant classification tasks while grouping batch correction benchmarks as a separate evaluation axis, further highlighting the robustness of the model under reduced data scaling (S7 Table).

Finally, to assess generalization under cross-platform distribution shifts, we evaluated models on cardiomyocyte datasets spanning Drop-seq (single-cell) and DroNc-seq (single-nucleus) modalities [41]. Models were fine-tuned on single-cell data and evaluated in a zero-shot manner across modalities (Fig 3F). GF_CAB_ exhibited improved integration performance following fine-tuning, consistent with prior observations [19], further supporting its ability to generalize across technical and biological shifts.

Collectively, these results demonstrate that GF_CAB_ translates improved pretraining behavior into enhanced downstream performance, achieving competitive classification accuracy while consistently improving generalization in zero-shot settings. Importantly, these gains are maintained, and in some cases amplified, under reduced data scaling, highlighting that principled architectural alignment can preserve and even enhance the generalization capacity of single-cell foundation models without reliance on large-scale pretraining.

### Component-wise contributions of GF_CAB_ and benchmarking against foundation models

**Fig 4:**
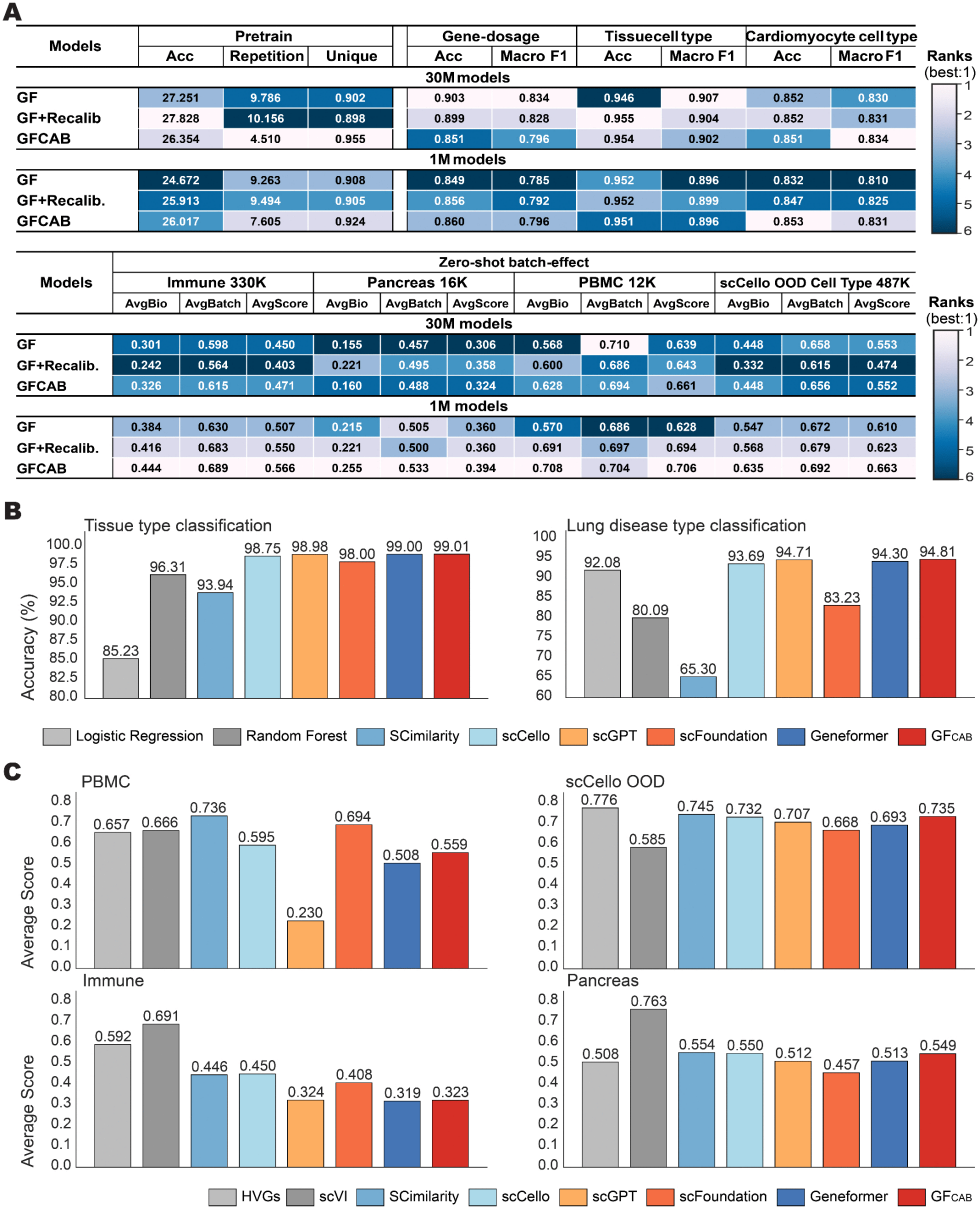
Ablation and benchmarking of GF_CAB_ across downstream classification and zero-shot batch integration tasks. **(A)** Ablation analysis of GF_CAB_, evaluating the contributions of its components across three classification tasks (gene dosage sensitivity, cardiomyocyte disease-state classification, and spleen cell type classification) and four zero-shot batch integration benchmarks. Numerical performance metrics are shown alongside color-coded rankings. **(B)** Classification accuracy benchmarking of GF_CAB_ against classical baselines (logistic regression, random forest) and foundation model counterparts (scCello, scGPT, scFoundation, SCimilarity, and GF) under a unified data processing and evaluation framework, across tissue type classification and lung disease classification tasks. **(C)** Zero-shot batch integration performance, reported as average scores (0.6×AvgBIO + 0.4×AvgBatch), comparing GF_CAB_ with representative methods (highly variable genes (HVGs), scVI) and foundation models (scCello, scGPT, scFoundation, SCimilarity, and GF) across four datasets under the same evaluation pipeline.

To systematically dissect the contributions of the cumulative-assignment recalibration and similarity-based regularization in the CAB module, we conducted an ablation study comparing three model variants: the original Geneformer (GF), GF with cumulative-assignment recalibration only (GF+Recalib.), and the full GF_CAB_ model. These variants were evaluated across pretraining behavior, downstream classification, and zero-shot batch integration tasks under both 1M and 30M data scales (Fig 4A, S8 Table).

For masked gene prediction, the combination of cumulative-assignment and similarity-based regularization consistently improved both accuracy and predictive diversity relative to GF. In contrast, cumulative-assignment alone yielded limited gains and, in some cases, slightly underperformed GF, suggesting that suppressing redundancy without additional distributional regularization is insufficient to fully improve predictive behavior. The full GF_CAB_ model, however, achieved stable improvements across scales, highlighting the complementary roles of the two components in shaping well-calibrated and diverse prediction distributions.

In downstream classification tasks, both GF+Recalib. and GF_CAB_ generally matched or exceeded the performance of GF, although gains were task-dependent. The addition of similarity-based regularization did not consistently increase classification accuracy beyond cumulative reassignment alone but preserved comparable performance while enhancing prediction diversity (S8 Table). This indicates that the similarity constraint acts primarily as a regularizing factor, reducing redundancy without compromising discriminative capacity. Notably, given the already strong baseline performance of GF, these improvements are incremental but consistent.

The most pronounced improvements were observed in zero-shot batch integration. Across all four datasets, both cumulative reassignment and similarity-based regularization progressively improved generalization compared to GF, with GF_CAB_ achieving the strongest overall performance (Fig 4A). These results indicate that enforcing cross-position constraints and distributional diversity can partially mitigate the known limitations of rank-based models in handling cross-dataset variability.

To contextualize these gains, we further benchmarked GF_CAB_ against other foundation models, including scCello, scGPT, scFoundation, SCimilarity, and GF, under a unified data processing and evaluation framework (Fig 4, S6 Fig) [4,15,35,42]. Across classification tasks (tissue type and lung disease classification), GF_CAB_ achieved the highest accuracy, further extending the strong discriminative performance of ranking-based model GF (Fig 4B). These results reinforce that architectural alignment within the rank-based modeling paradigm can yield state-of-the-art performance for classification tasks.

We next extended this comparison to zero-shot batch integration (Fig 4C). Consistent with prior studies [35,39,40,43], ranking-based foundation models, including GF and GF_CAB_, remain less competitive than dedicated integration methods such as HVG-based representations and scVI. Among foundation models, scFoundation and SCimilarity, both characterized by relatively simpler architectures, exhibited stronger generalization, while scCello outperformed GF by incorporating cell ontology information to guide representation learning. In this context, GF_CAB_ consistently improved over GF within the naive ranking-based family, but did not fully close the performance gap with non-ranking-based approaches. This suggests that while architectural refinement can alleviate some limitations, intrinsic constraints of rank-based representations remain a key factor in zero-shot generalization.

Collectively, these results demonstrate that the CAB module provides complementary improvements in predictive behavior and downstream performance. While these gains further strengthen the already high discriminative capacity of ranking-based foundation models, they also highlight the persistent gap in zero-shot batch integration, underscoring the need for future advances beyond architectural refinement alone.

## Discussion

Model architectures that are explicitly aligned with the structural properties of single-cell transcriptomic data can yield consistent and meaningful performance gains. In this work, GF_CAB_ addresses a fundamental limitation of rank-based modeling by mitigating repetitive predictions in masked gene recovery, while improving overall predictive accuracy and sensitivity to biologically relevant signals. By integrating cumulative-assignment recalibration and similarity-based regularization, GF_CAB_ produces more diverse and well-calibrated predictions, enabling the recovery of low-prevalence genes with functional significance. These findings demonstrate that architectural alignment with the rank-ordered nature of gene expression profiles can offset, and in some cases surpass, the benefits of large-scale pretraining, highlighting model design as a critical determinant of performance.

A second key contribution of this work is the systematic evaluation of data scaling in single-cell foundation models. Contrary to the prevailing assumption that larger pretraining datasets uniformly improve performance, we show that gains in both predictive behavior and downstream tasks exhibit clear diminishing returns, with the majority of improvements occurring early in training. Notably, under an improved architecture such as GF_CAB_, models pretrained on substantially smaller datasets can match or exceed the performance of models trained on datasets up to 30-fold larger. Furthermore, smaller-scale models consistently exhibit stronger generalization in zero-shot settings, suggesting that excessive scaling may reinforce dataset-specific biases at the expense of transferability.

Collectively, this study addresses two underexplored challenges in the development of single-cell foundation models: the mismatch between model architectures and rank-ordered transcriptomic representations, and the limited understanding of data scaling effects in this domain. Our results motivate a shift in design principles, emphasizing that scaling alone is insufficient without architectures that respect the intrinsic structure of biological data. While rank-based representations provide a powerful framework for capturing gene expression patterns, they also impose inherent constraints, particularly in settings requiring robust cross-dataset generalization. Future progress will likely depend on jointly advancing model architectures and representation strategies to better reconcile predictive accuracy, diversity, and generalization in single-cell learning systems.

## Methods

### Datasets

For full-scale pre-training, we followed the protocol established in Geneformer (GF) and used the Genecorpus-30M dataset [19], which contains 29,900,531 single-cell transcriptomic profiles, where the genes in each cell were ranked by normalized expression values. To examine the impact of reduced data scale, we constructed Genecorpus-1M by randomly sampling one million profiles from the original corpus. In addition, we curated a non-overlapping dataset of five million profiles (Genecorpus-5M), which was reserved exclusively for the unbiased evaluation of the masked pretraining objective.

Downstream benchmarking relied on datasets that were previously used in the GF study to ensure comparability across tasks. The gene dosage-sensitivity task was defined using 122 dosage-sensitive and 368 dosage-insensitive genes reported in previous studies [44–46], along with 50,000 ranked profiles curated by Theodoris et al. [19] (Genecorpus gene dosage 50K). Multi-tissue cell type classification was performed on 249,556 profiles spanning 59 cell types across nine tissues (spleen, kidney, lung, liver, brain, placenta, large intestine, immune system, and pancreas) [19] (Genecorpus cell type 250K). For cardiomyocyte disease-state classification, we used the profiles from Chaffin et al. [47], which included cardiomyocytes from non-failing hearts, as well as from patients with hypertrophic cardiomyopathy (HCM) [48] and dilated cardiomyopathy (DCM) [49]. This dataset encompassed 580,000 ranked profiles from 42 individuals, annotated with metadata including age, sex, and cell type, producing a three-class classification task (Chaffin cardiomyocyte 580K). To evaluate the finetuned batch effect integration of pretrained models on biologically relevant dataset, we conducted research on the dual platform sequencing dataset on cardiomyocyte [41], with both the Drop-seq (single-cell) sequencing data and the DorNc-seq (single-nucleus) data.

To evaluate the quality of learned cell embeddings and assess batch integration under zero-shot conditions, we incorporated four external datasets following prior work [35,39]: Pancreas 16K [33], PBMC 12K [12,34], Immune 330K [32], and scCello OOD cell type 487K [35].

The expanded benchmark includes two classification tasks and zero-shot batch integration across four datasets. The classification tasks consist of (i) a multi-tissue classification task using eight tissues (bone marrow, eye, heart, kidney, large intestine, small intestine, liver, and muscle) from the Tabula Sapiens dataset [50], and (ii) a lung disease classification task (COPD, IPF, control) using dataset GSE136831 [51]. For batch integration, we evaluated models on Pancreas 16K [33], PBMC 12K [12,34], Immune 330K [32], and the scCello OOD dataset (487K cells) [35] with re-processing from scratch to synchronize evaluation frameworks.

### Enhancing prediction diversity with GF_CAB_

Geneformer pioneered the application of language models (LMs) for single-cell RNA-seq by representing gene co-expression patterns as rank-ordered sequences [19]. Although effective, its masked language modeling (MLM) framework exhibits three limitations: (1) repeated assignment of the same gene across multiple masked positions within a profile, (2) systematic under-representation of low-expression genes, and (3) frequency-driven bias toward highly expressed, ubiquitous genes. To address these issues, we propose **GF**_**CAB**_ (Geneformer with **C**umulative-assignment on the prediction **A**djustment and **B**oosting on the similarity-based diversity), which augments GF with two complementary mechanisms: cumulative-assignment prediction adjustment and similarity-based regularization.

#### Cumulative-assignment prediction adjustment.

In standard MLM, masked positions are predicted independently, allowing the same gene to be assigned repeatedly across multiple sites. To mitigate this redundancy, we introduce a cumulative assignment-and-blocking mechanism that integrates one-hot substitution with cumulative Noisy-OR suppression [24].

Given a masked sequence ${\tilde{C}}_{i}=[g_{1}^{i},\;\text{<MASK>},\;g_{3}^{i},\;...,\;\text{<MASK>},\;...,\;g_{k}^{i}]$ of *k* tokens, the BERT model produces a k×V probability matrix $P_{i}$, where V∈ℝ is the vocabulary size. Observed positions are replaced with one-hot vectors: $P_{i}^{\prime }(j,g)=1$ if $g=g_{j}^{i}$, and 0 otherwise. A blocking vector $B_{i}\in \{0,1{\}}^{V}$ is then defined such that $B_{i}(g)=1$ if $g\in G_{\text{observed}}$ (observed gene set within a profile), and it is concatenated with $P_{i}^{\prime }$ to form the augmented matrix $\bar{P_{i}}$, as shown in Eq (1).


$$\begin{array}{c} \bar{P_{i}}=[\begin{array}{c} B_{i}\\ P_{i}^{\prime } \end{array}]\in {\mathbb{R}}^{(k+1)\times V}\;. \end{array}$$ (1)

After complement ${\bar{P}}_{i}^{\prime }=1-{\bar{P}}_{i}$, Noisy-OR normalization produces adjusted probabilities ${\tilde{P}}_{i}(j,g)$, as shown in Eq (2), which enforces cumulative suppression across positions. This mechanism mitigates duplicate predictions within the same cell profile, thereby improving biological plausibility.


$$\begin{array}{c} {\tilde{P}}_{i}(j,g)={\bar{P}}_{i}(j,g)\cdot \prod_{s<j}{\bar{P}}_{i}^{\prime }(s,g)\;. \end{array}$$ (2)

#### Similarity-based regularization.

Despite the redundancy observed within individual cell profiles, the predictions made by the GF remain strongly biased toward highly expressed, ubiquitous genes. This pattern suggests an overreliance on global expression trends, with limited utilization of local, context-specific signals that are essential for meaningful biological interpretation.

To address this frequency bias limitation, we introduce a similarity-based regularization strategy, inspired by Ayinde *et al.* [52], which is designed to penalize overly similar predictions across masked positions and encourage context-aware inference. This approach directly counteracts the model’s tendency to favor frequent and highly expressed genes, thus improving its ability to capture rare, condition-specific signals and consequently improving predictive diversity.

For masked positions $\left(j_{m},q_{m}\right)$ in sequence ${\tilde{C}}_{i}$, we compute pairwise similarity between adjusted distributions, as shown in Eq (3).


$$\begin{array}{c} sim({\tilde{P}}_{i}(j_{m}),{\tilde{P}}_{i}(q_{m}))={\tilde{P}}_{i}(j_{m})\cdot {\tilde{P}}_{i}(q_{m})\;. \end{array}$$ (3)

Summing across all $N_{mp}$ distinct masked-pair combinations produces a diversity loss, Eq (4), which discourages uniform predictions across masked sites by penalizing distributional redundancy.


$$\begin{array}{c} L_{D_{i}}=\sum_{j_{m}\neq q_{m}}sim({\tilde{P}}_{i}(j_{m}),{\tilde{P}}_{i}(q_{m}))\;. \end{array}$$ (4)

Finally, we incorporate this similarity penalty into the masked sequence pretraining objective Eq (5) by combining it with the standard cross-entropy loss. Here, S∈ℝ, $N_{m}\in \mathbb{R}$, and $y_{i}\in {\mathbb{R}}^{V}$ denote the number of sequences, the number of masked positions, and the one-hot representation of the ground-truth label for sequence *i*, respectively. The hyperparameter λ∈ℝ controls the strength of diversity regularization. This penalty discourages over-concentration on frequent genes, promoting rare and context-sensitive predictions that are critical for biological discovery.


$$\begin{array}{c} ℒ=-\frac{1}{N_{m}}\sum_{i=1}^{S}y_{i}\cdot \log{\tilde{P}}_{i}+\lambda \cdot \frac{1}{N_{mp}}\sum_{i=1}^{S}L_{D_{i}}\;. \end{array}$$ (5)

### Baseline models

We compared GF_CAB_ against GF in three dimensions: pre-training objectives, downstream classification, and zero-shot batch-effect mitigation. In classification tasks, we also included Support Vector Machine (SVM) [38], Random Forest (RF) [37], and Logistic Regression (LR) as baselines. For zero-shot settings, GF and GF_CAB_ were compared with highly variable genes (HVG) and scVI [53], a variational autoencoder framework that explicitly models batch effects.

### Evaluation metrics

For pretraining evaluation, models were evaluated on the masked gene prediction objective using metrics that jointly capture predictive accuracy and diversity. Masked prediction accuracy quantifies the proportion of correctly reconstructed masked tokens across the validation set, reflecting the model’s ability to recover contextually appropriate genes. Repetition ratios measure redundancy in predictions and are reported in three forms: the overall repetition ratio (fraction of all duplicate predictions), the unmasked repetition ratio (proportion of predictions overlapping with unmasked input tokens), and the masked repetition ratio (fraction of repeated predictions restricted to masked positions). To further characterize predictive diversity, we introduced a uniqueness score, inspired by entropy-based measures such as the Kullback–Leibler divergence [54]. This score reflects the proportion of distinct gene predictions at masked sites, capturing the model’s ability to avoid mode collapse and explore less frequent, yet biologically informative genes. Higher uniqueness values indicate more diverse and context-sensitive predictions.

For downstream tasks, we used classification, clustering, and batch integration metrics to jointly assess biological relevance and technical robustness. Classification performance was evaluated by accuracy, macro-averaged AUC [55,56], macro and weighted F1 scores [57,58], and recall [59], complemented by confusion matrices for error-structure visualization [60]. Cluster quality, following established benchmarking frameworks [33,39], was quantified using normalized mutual information (NMI) [61], adjusted Rand index (ARI) [62], and average silhouette width (ASW_label_), which were aggregated into the AvgBIO metric [4] to summarize cluster separability. Batch integration was assessed through the average silhouette width with respect to batch (ASW_batch_) [63] and graph connectivity (GraphCon) [35], which were combined into an AvgBatch measure. To provide a balanced evaluation, an overall score (AvgScore) [4] was reported, defined as the mean of AvgBIO and AvgBatch [35]. For benchmarking against other foundation models, AvgScore was alternatively computed as 0.6×AvgBIO + 0.4×AvgBatch to align with the practice of Kedzierska et al. [39], thereby integrating biological fidelity with batch mixing quality.

### Implementation details

We implemented the proposed architecture, GF_CAB_, as an extension of the masked language model GF. The model retains GF’s core transformer design (6 encoder layers, 4 attention heads, 256-dimensional embeddings, 512-dimensional feedforward layers, and a maximum input length of 2,048) while introducing cumulative prediction adjustment and a similarity-based loss penalty for diversity boosting. Hyperparameters, training schedules, and preprocessing strategies were aligned with GF to enable direct comparison (see S9 Table). Input preparation followed the standard masking scheme through a modified Hugging Face Trainer framework [64], with 15% of tokens selected for perturbation and custom batching to account for variable gene counts per cell. Pre-training was conducted at two scales: a 1M-cell dataset (Genecorpus-1M) with a batch size of 12 for rapid iteration and a 30M-cell dataset (Genecorpus-30M) with a batch size of 8 for full-scale training, both run on 8 NVIDIA A100 GPUs. GF was pretrained under identical conditions to ensure a controlled baseline.

For downstream evaluation, we fine-tuned GF, GF_CAB_, and classical machine learning baselines (SVM, random forest, and logistic regression) on three classification tasks that reflect distinct biological challenges. Depending on task requirements, we used either Hugging Face’s BertForTokenClassification for gene-level prediction or BertForSequenceClassification for cell profile–level tasks, initializing with pre-trained weights and applying GF’s fine-tuning conventions. Hyperparameters such as frozen layers, learning rate schedules, warmup steps, and epochs were set according to the GF’s released scripts, with minor task-specific adjustments (*e.g.*, different frozen layer numbers and learning rates for cardiomyocyte classification). Baseline classifiers were implemented using scikit-learn [65] with default parameters, except for training epochs, which were harmonized with task objectives.

To test zero-shot transferability, we compared the embeddings from GF_CAB_ and GF with those derived from highly variable genes (HVG) and scVI, following the protocol established by Kedzierska *et al.* [39]. For the transformer models, cell embeddings were computed from unmasked gene inputs without further masking or fine-tuning, ensuring strict zero-shot conditions. HVG features provided a conventional dimensionality reduction baseline, while scVI embeddings were drawn directly from the latent space. All embeddings were benchmarked using cluster- and integration-based metrics, allowing for a direct comparison between foundation models and established single-cell representation methods.

### Statistical analysis

Pre-training results are reported as the mean ± standard deviation across 50,000 independent ranked single-cell profiles. For downstream tasks, accuracy and macro-F1 values were averaged over 10 independent cross-validation runs (where applicable). Statistical significance of performance differences was evaluated using the paired Wilcoxon signed-rank test, with each model compared pairwise against GF-30M as the baseline, unless otherwise noted.

### Reproducibility

All experiments were performed using PyTorch (v2.6.0) with Hugging Face Transformers (v4.38.2), CUDA (v11.8), and Python (v3.11.7). Training was distributed across 8 NVIDIA A100 GPUs with mixed precision enabled. Random seeds were fixed across NumPy, PyTorch, and CUDA to ensure replicability. The complete codebase, pretrained weights, evaluation scripts, and datasets incorporated in this study are available at the following repositories.

## Supporting information

S1 TablePretraining performance of models on 50,000 profiles from Genecorpus-5M.Pretraining results are reported for Geneformer and GF_CAB_ models with varying similarity regularization coefficients (λ), trained on different scales of pretraining data and evaluated on 50,000 test profiles randomly sampled from Genecorpus-5M. Metrics include masked prediction accuracy (mean ± standard deviation), repetition ratios (overall, unmasked, and masked), and uniqueness scores.(XLSX)

S2 TableGene dosage-sensitivity prediction performance of Geneformer and GF_CAB_ models.Macro F1-scores and accuracies are reported for Geneformer and GF_CAB_ variants with different similarity regularization coefficients (λ), pretrained on either Genecorpus-30M or Genecorpus-1M. Bold values indicate the best performance, while underlined values indicate the second-best performance.(XLSX)

S3 TableCardiomyocyte cell type prediction performance of Geneformer and GF_CAB_ models.Macro F1-scores and accuracies are reported for Geneformer and GF_CAB_ variants with different similarity regularization coefficients (λ), pretrained on either Genecorpus-30M or Genecorpus-1M. Bold values indicate the best performance, while underlined values indicate the second-best performance.(XLSX)

S4 TableTissue-specific cell type prediction performance of Geneformer and GF_CAB_ models.Macro F1-scores and accuracies are reported for individual tissues using Geneformer and GF_CAB_ variants with different similarity regularization coefficients (λ), pretrained on either Genecorpus-30M or Genecorpus-1M. Bold values denote the best performance, while underlined values denote the second-best performance.(XLSX)

S5 TableSpleen-specific cell type prediction performance of Geneformer and GF_CAB_ models.Macro F1-scores and accuracies are reported for spleen-derived cells using Geneformer and GF_CAB_ variants with different similarity regularization coefficients (λ), pretrained on either Genecorpus-30M or Genecorpus-1M. Bold values denote the best performance, while underlined values denote the second-best performance.(XLSX)

S6 TableZero-shot batch-effect mitigation performance of Geneformer and GF_CAB_ models.Batch-effect mitigation performance is reported for Geneformer and GF_CAB_ variants with different similarity regularization coefficients (λ), pretrained on either Genecorpus-30M or Genecorpus-1M. Evaluation metrics include cell type clustering measures (NMI, ARI, ASW_label_, AvgBio), batch clustering measures (Graph connectivity score, ASW_batch_, AvgBatch), and the overall score (average of AvgBio and AvgBatch).(XLSX)

S7 Table Ranking results across three classification tasks and zero-shot batch integration evaluated on four datasets. Overall rankings are computed using two strategies: (i) treating each batch integration dataset as an independent task; and (ii) aggregating all batch integration results into a single task before averaging with the three classification tasks. Across both strategies, the smaller-scale pretrained GF_CAB_ model achieves the strongest overall performance, supporting the conclusion that a reduced pretraining scale combined with the proposed architectural modifications can maintain competitive performance while improving generalization to unseen datasets.
(XLSX)

S8 TableAblation study results for Geneformer and GF_CAB_ models.Ablation results are reported for Geneformer and GF_CAB_ variants with different similarity regularization coefficients (λ), pretrained on either Genecorpus-30M or Genecorpus-1M. Evaluations span pretraining performance, gene dosage-sensitivity prediction, tissue-specific and cardiomyocyte cell type classification, and zero-shot batch-effect mitigation across four datasets using the metrics described above. Bold values denote the best performance, while underlined values denote the second-best performance.(XLSX)

S9 Table Pretraining hyperparameters for Geneformer and GF_CAB_ models. Detailed hyperparameter configurations used for Geneformer and GF_CAB_ models benchmarked in this study are reported.
(XLSX)

S1 Fig Comparison between elongating training on 1M data and early stopping on 30M data to investigate the net effect brought by the remaining 29M data. (A) Pretraining masked token prediction accuracy, repetition ratios, and uniqueness across different data scales and different training steps. (B) Tissue type classification accuracy, macro F1 score, and weighted F1 score across data scales and different training steps.
(PDF)

S2 Fig Scaling behavior of GF and GF_CAB_ across different pretraining data sizes (10K, 100K, 1M, and 30M cells). (A) Pretraining performance, including masked token prediction accuracy, repetition ratio, and uniqueness. (B) Downstream tissue-type classification performance, measured by accuracy, macro F1, and weighted F1 scores. Both models show performance improvements with increasing data scale, with diminishing returns observed beyond 1M cells.
(PDF)

S3 Fig Effect of model scaling on performance for GF and GF_CAB_. Model scale is measured by the number of trainable parameters (ranging from 4.35M to 26.97M). (A) Pretraining performance, including masked token prediction accuracy, repetition ratio, and uniqueness. (B) Tissue-type classification performance (accuracy, macro F1, weighted F1). (C) Lung disease classification performance (COPD, IPF, control; accuracy, macro F1, weighted F1). Moderate increases in model size improve performance, while excessive scaling leads to diminishing or negative returns, likely due to data–model mismatch. GF_CAB_ consistently outperforms GF across all model scales.
(PDF)

S4 Fig Comparison between original model predictions and predictions under an enforced-uniqueness constraint across masked positions. (A) Cross-model and cross-scale comparison showing prediction accuracy for Geneformer and GF_CAB_ variants trained on different data scales. (B) Direct comparison of Geneformer and GF_CAB_ (1M and 30M pretraining scales), illustrating the impact of enforcing uniqueness on prediction accuracy. Enforcing uniqueness eliminates repetition (repetition ratio = 0) but leads to a noticeable accuracy drop, particularly for Geneformer, whereas GF_CAB_ demonstrates improved robustness under this constraint.
(PDF)

S5 Fig Characterization of low-frequency genes uniquely recovered by GF_CAB_. (A) Distribution of sequence-level occurrence, measured as the proportion of sequences in Genecorpus-30M containing each gene uniquely predicted by GF_CAB_. (B) Distribution of overall gene frequency in Genecorpus-30M for these genes, demonstrating their low prevalence in the pretraining dataset. (C) Representative examples of biologically relevant genes recovered by GF_CAB_ but not by Geneformer, including lineage-defining transcription factors (ASCL1, OLIG2, KLK6), tissue-specific markers (BGLAP, GATA4), and disease-relevant signaling and remodeling mediators (FGFR3, MMP9). (D) Comparison of the frequency distribution of low-prevalence genes predicted by Geneformer and GF_CAB_, showing that GF_CAB_ preferentially captures genes with lower dataset frequency, consistent with enhanced predictive diversity.
(PDF)

S6 FigBenchmark comparison of rank-based and non–rank-based foundation models under a unified evaluation framework.(A) Tissue classification performance (accuracy and macro F1) across eight tissues from Tabula Sapiens. (B) Lung disease classification performance (COPD, IPF, control) evaluated using dataset GSE136831. (C) Zero-shot batch integration performance measured by AvgScore (0.6×AvgBio + 0.4×AvgBatch) across four datasets (Pancreas 16K, PBMC 12K, Immune 330K, and scCello OOD). Models include GF, GF_CAB_, scGPT, scFoundation, SCimilarity, and scCello, with classical baselines (logistic regression and random forest) for classification and HVG-based representation and scVI for batch integration. GF_CAB_ consistently improves over GF while remaining competitive within the rank-based modeling paradigm.(PDF)
