# Supplementary material for: Assessing scale and predictive diversity in models for single-cell transcriptomics based on Geneformer
Source: PLoS Comput Biol. 2026 Jul 30;22(7):e1013701. doi: 10.1371/journal.pcbi.1013701 (PMC13456263; doi:10.1371/journal.pcbi.1013701)
Supplement: Supplementary file 2 [file pcbi.1013701.s011.pdf]

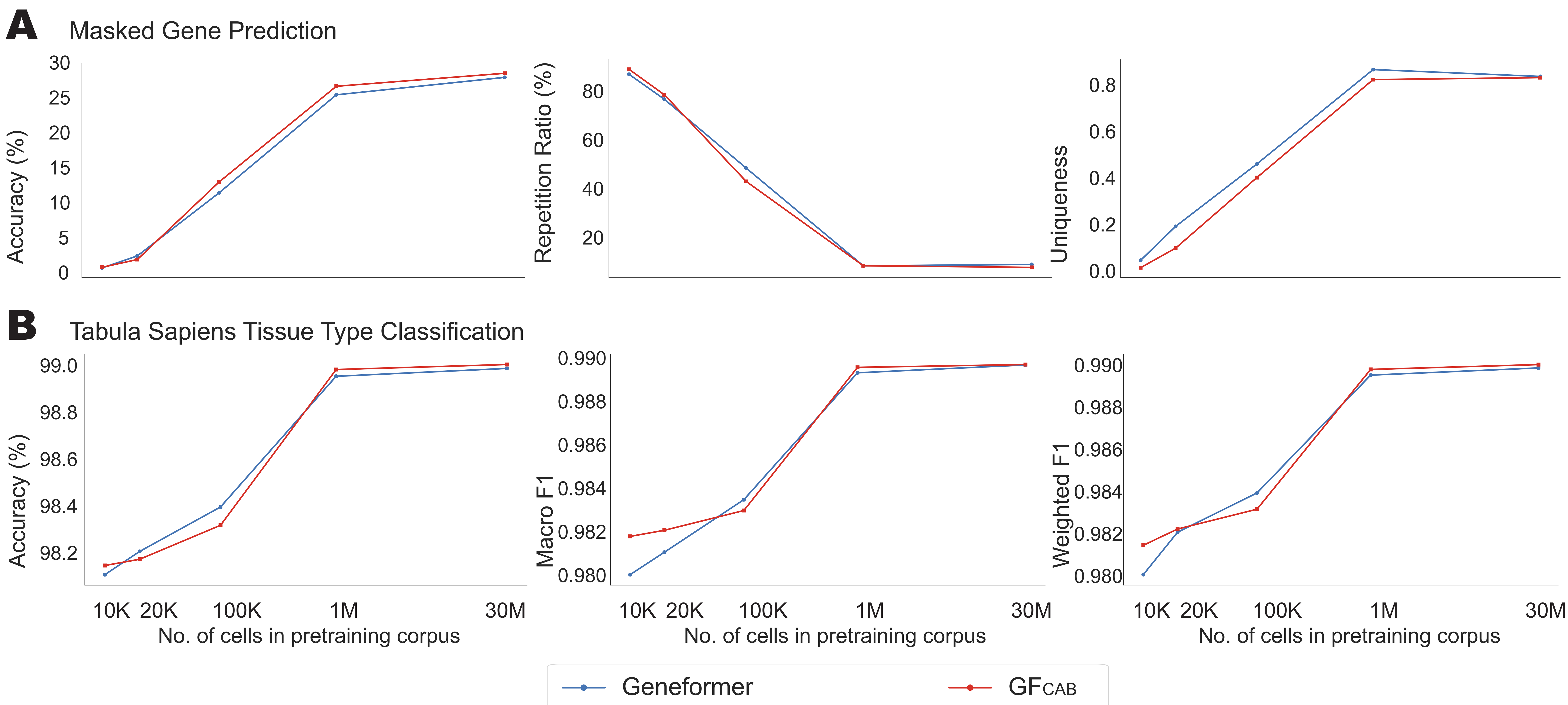
A
Masked Gene Prediction
Accuracy (%)
Repetition Ratio (%)
Uniqueness
B
Tabula Sapiens Tissue Type Classification
Accuracy (%)
Macro F1
Weighted F1
10K 20K 100K 1M 30M
No. of cells in pretraining corpus
Geneformer
GFCAB
