# Supplementary material for: Assessing scale and predictive diversity in models for single-cell transcriptomics based on Geneformer
Source: PLoS Comput Biol. 2026 Jul 30;22(7):e1013701. doi: 10.1371/journal.pcbi.1013701 (PMC13456263; doi:10.1371/journal.pcbi.1013701)
Supplement: Supplementary file 4 [file pcbi.1013701.s013.pdf]

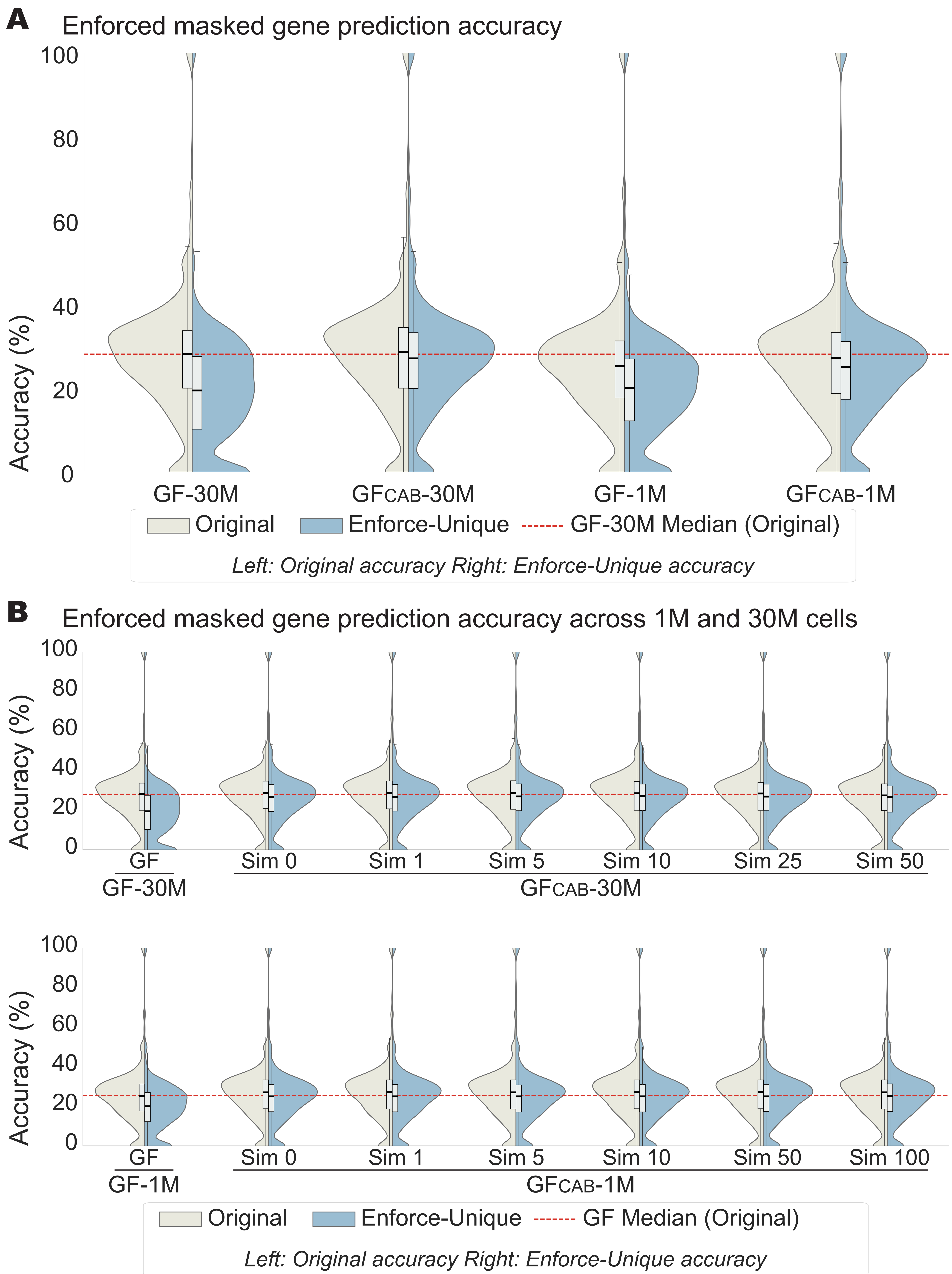

A
Enforced masked gene prediction accuracy
Accuracy (%)
100
80
60
40
20
0
GF-30M
GFCAB-30M
GF-1M
GFCAB-1M
Original
Enforce-Unique
GF-30M Median (Original)
Left: Original accuracy Right: Enforce-Unique accuracy
B
Enforced masked gene prediction accuracy across 1M and 30M cells
Accuracy (%)
GF
GF-30M
Sim 0
Sim 1
Sim 5
Sim 10
Sim 25
Sim 50
GFCAB-30M
GF
GF-1M
Sim 0
Sim 1
Sim 5
Sim 10
Sim 50
Sim 100
GFCAB-1M
Original
Enforce-Unique
GF Median (Original)
Left: Original accuracy Right: Enforce-Unique accuracy
