# Supplementary material for: Assessing scale and predictive diversity in models for single-cell transcriptomics based on Geneformer
Source: PLoS Comput Biol. 2026 Jul 30;22(7):e1013701. doi: 10.1371/journal.pcbi.1013701 (PMC13456263; doi:10.1371/journal.pcbi.1013701)
Supplement: Supplementary file 5 [file pcbi.1013701.s014.pdf]

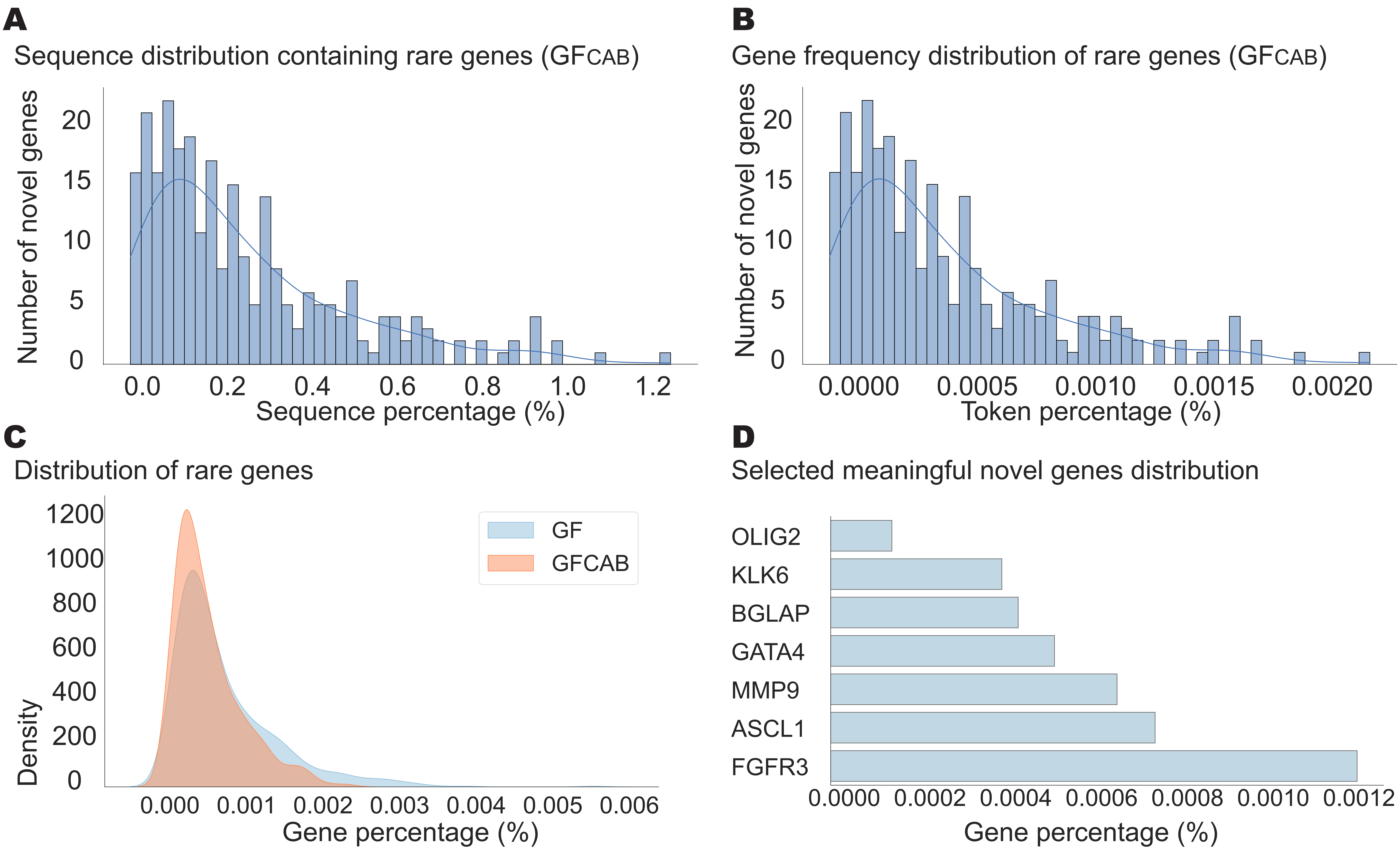
A
Sequence distribution containing rare genes (GFCAB)
Number of novel genes
Sequence percentage (%)
B
Gene frequency distribution of rare genes (GFCAB)
Number of novel genes
Token percentage (%)
C
Distribution of rare genes
GF
GFCAB
Density
Gene percentage (%)
D
Selected meaningful novel genes distribution
OLIG2
KLK6
BGLAP
GATA4
MMP9
ASCL1
FGFR3
Gene percentage (%)
