# Supplementary material for: Assessing scale and predictive diversity in models for single-cell transcriptomics based on Geneformer
Source: PLoS Comput Biol. 2026 Jul 30;22(7):e1013701. doi: 10.1371/journal.pcbi.1013701 (PMC13456263; doi:10.1371/journal.pcbi.1013701)
Supplement: Supplementary file 6 [file pcbi.1013701.s015.pdf]

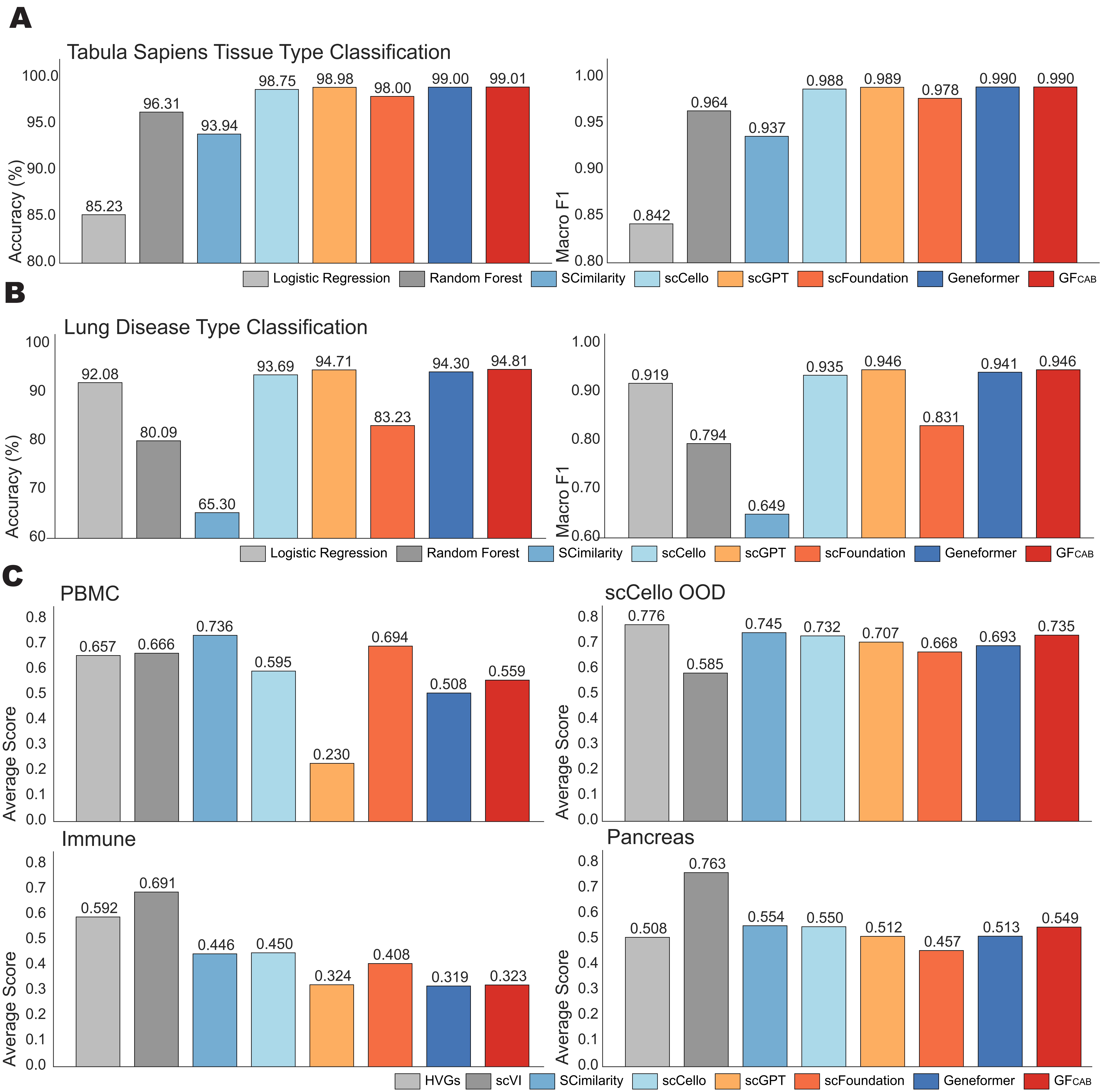

A
Tabula Sapiens Tissue Type Classification
Accuracy (%)
85.23
96.31
93.94
98.75
98.98
98.00
99.00
99.01
Macro F1
0.842
0.964
0.937
0.988
0.989
0.978
0.990
0.990
Logistic Regression
Random Forest
SCimilarity
scCello
scGPT
scFoundation
Geneformer
GFCAB
B
Lung Disease Type Classification
Accuracy (%)
92.08
80.09
65.30
93.69
94.71
83.23
94.30
94.81
Macro F1
0.919
0.794
0.649
0.935
0.946
0.831
0.941
0.946
C
PBMC
Average Score
0.657
0.666
0.736
0.595
0.230
0.694
0.508
0.559
scCello OOD
0.776
0.585
0.745
0.732
0.707
0.668
0.693
0.735
Immune
0.592
0.691
0.446
0.450
0.324
0.408
0.319
0.323
Pancreas
0.508
0.763
0.554
0.550
0.512
0.457
0.513
0.549
HVGs
scVI
SCimilarity
scCello
scGPT
scFoundation
Geneformer
GFCAB
