# Supplementary material for: Assessing scale and predictive diversity in models for single-cell transcriptomics based on Geneformer
Source: PLoS Comput Biol. 2026 Jul 30;22(7):e1013701. doi: 10.1371/journal.pcbi.1013701 (PMC13456263; doi:10.1371/journal.pcbi.1013701)
Supplement: Supplementary file 7 [file pcbi.1013701.s017.pdf]

Response Letter

The authors sincerely thank the editor and reviewers for their constructive comments and suggestions, which have substantially improved the quality of this manuscript. The manuscript has been carefully revised in response to all comments, and our detailed responses are provided below. For clarity, responses and significant changes in the revised manuscript are highlighted in blue with the location (line numbers) of the changes in the revision indicated at the end of each response.

We also appreciate the reviewers' thoughtful insights and thorough evaluation of our work. Their feedback has motivated additional analyses and figure revisions, leading to new results and improved presentation. To fully address the points raised, we have substantially revised and reorganized the manuscript to incorporate these additions and to more clearly present the key contributions.

Response to the reviewers

Reviewer 1

Summary from the reviewer:

The manuscript evaluates how pre-training scale and architectural adjustments influence the performance of Geneformer-based foundation models for single-cell transcriptomics. The authors introduce the GF-CAB variant and benchmark both Geneformer and GF-CAB across masked gene prediction, several classification tasks, and zero-shot batch-effect mitigation. The work addresses a relevant topic: the growing assumption that models trained on larger datasets will improve downstream performance.

Major comments

Reviewer Comment 1.1 — The cumulative-assignment and similarity-based regularization components are reasonable attempts to address a known issue: repetition of the same gene across multiple masked positions. The manuscript would benefit from a clearer comparison to simpler alternatives. For example, would re-assigning a fixed "first rank position" to each gene and enforcing unique predictions across positions achieve similar redundancy reduction?

Reply:

We thank the reviewer for this insightful suggestion. To directly address this point, we conducted an additional experiment comparing the original masked token prediction strategy with a simplified alternative that enforces uniqueness across masked positions.

Specifically, under the uniqueness-enforcing setting, once a gene has been predicted at a preceding masked position, it is excluded from subsequent predictions, and the next highest-probability gene is selected instead. This procedure guarantees that each position receives a unique prediction, thereby eliminating redundancy (*i.e.*, repetition ratio = 0).

We evaluated this strategy on both the baseline Geneformer models and our GF_{CAB} variants across multiple pretraining data scales. The results are summarized in Fig 1. Enforcing uniqueness consistently reduces prediction accuracy compared to the models' original outputs, indicating that naive post hoc constraints disrupt the model's learned probability structure. This effect is particularly pronounced for Geneformer, where substantial performance degradation is observed across different data scales.

In contrast, GF_{CAB} models exhibit markedly improved robustness under the same constraint, with only minor reductions in accuracy. We attribute this resilience to the cumulative-assignment and similarity-based regularization components, which explicitly discourage redundant predictions during training rather than relying on post hoc correction. Consequently, our approach achieves a similar objective: enhancing predictive diversity while preserving the integrity of the learned representation and maintaining higher accuracy.

Overall, these results suggest that while enforcing uniqueness at inference time can eliminate redundancy, it does so at the cost of predictive performance. In comparison, our method provides a principled, training-time solution that improves diversity without incurring substantial accuracy loss.

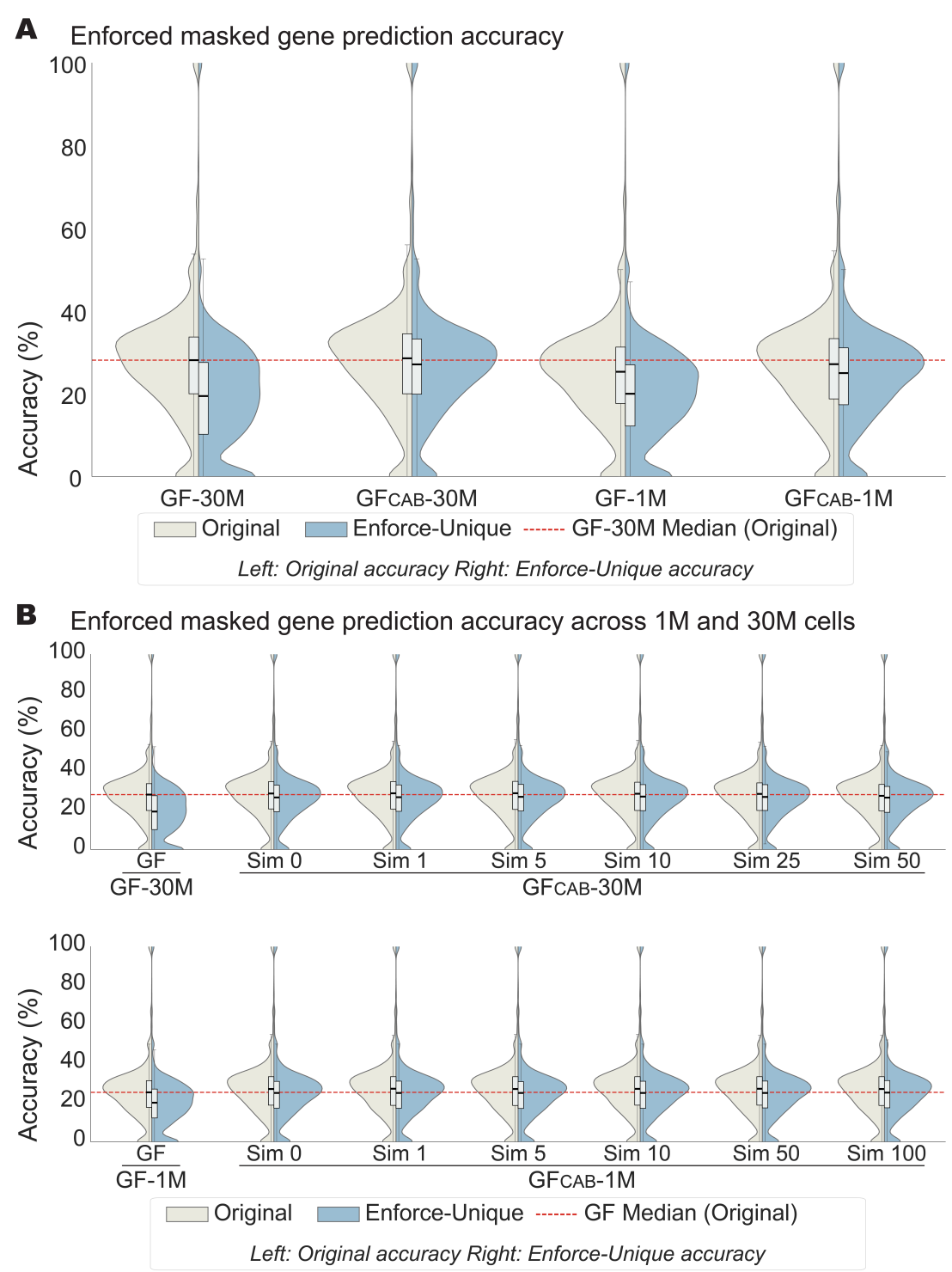

Figure 1: Comparison between original model predictions and predictions under an enforced-uniqueness constraint across masked positions. (A) Cross-model and cross-scale comparison showing prediction accuracy for Geneformer and GF_{CAB} variants trained on different data scales. (B) Direct comparison of Geneformer and GF_{CAB} (1M and 30M pretraining scales), illustrating the impact of enforcing uniqueness on prediction accuracy. Enforcing uniqueness eliminates repetition (repetition ratio $= 0$) but leads to a noticeable accuracy drop, particularly for Geneformer, whereas GF_{CAB} demonstrates improved robustness under this constraint.

Line number: 159-165.

Reviewer Comment 1.2 — The paper places substantial emphasis on repetition and uniqueness

as metrics of predictive diversity. However, the biological relevance of these changes is not explored. It would improve the manuscript if the authors examined which additional genes are recovered by GF-CAB (or by smaller-scale models) and whether these genes include rare markers, tissue-specific genes, or otherwise informative features.

Reply:

To evaluate the biological relevance associated with the reduction of repetition and enhancement of uniqueness in GF_{CAB}, we conducted an analysis on 10,000 randomly sampled test cell profiles. For each model, we collected all genes predicted at masked positions and compared the resulting gene sets.

We then identified genes uniquely predicted by GF_{CAB} but not by Geneformer. For these newly identified genes, we quantified their prevalence in the Genecorpus-30M pretraining dataset by examining (i) the proportion of sequences in which each gene appears and (ii) the overall gene frequency distribution (Fig 2A,B). The results indicate that the majority of these genes are low-frequency genes, characterized by limited presence across cells and low overall abundance in the dataset. Despite this, GF_{CAB} is able to recover and predict these genes, suggesting improved sensitivity to underrepresented biological signals.

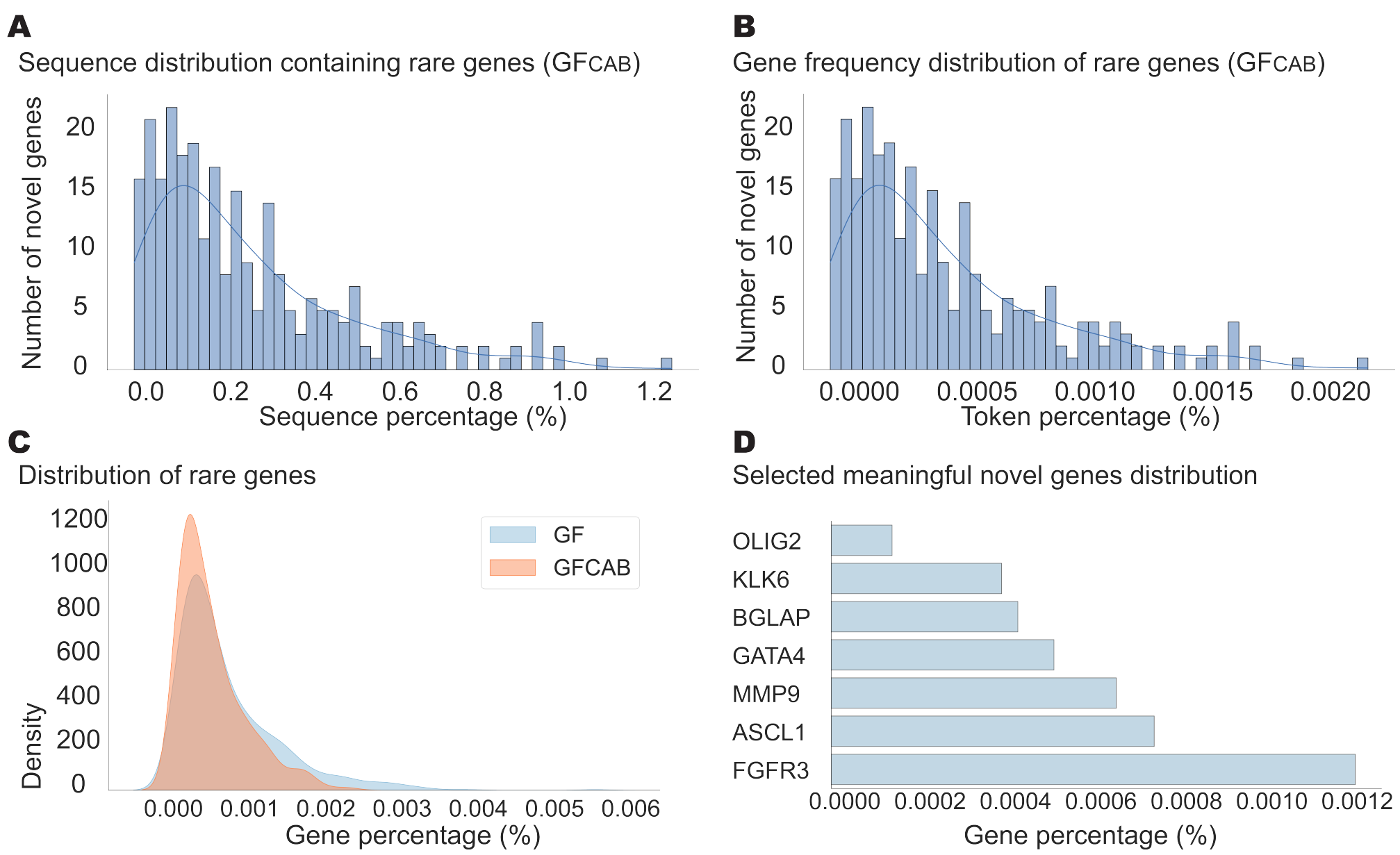

Figure 2: Characterization of low-frequency genes uniquely recovered by GF_{CAB}. (A) Distribution of sequence-level occurrence, measured as the proportion of sequences in Genecorpus-30M containing each gene uniquely predicted by GF_{CAB}. (B) Distribution of overall gene frequency in Genecorpus-30M for these genes, demonstrating their low prevalence in the pretraining dataset. (C) Representative examples of biologically relevant genes recovered by GF_{CAB} but not by Geneformer, including lineage-defining transcription factors (ASCL1, OLIG2, KLK6), tissue-specific markers (BGLAP, GATA4), and disease-relevant signaling and remodeling mediators (FGFR3, MMP9). (D) Comparison of the frequency distribution of low-prevalence genes predicted by Geneformer and GF_{CAB}, showing that GF_{CAB} preferentially captures genes with lower dataset frequency, consistent with enhanced predictive diversity.

To further assess biological relevance, we highlight seven representative genes: ASCL1, OLIG2, BGLAP, GATA4, FGFR3, MMP9, and KLK6, along with their corresponding frequency in Genecorpus-

30M (Fig 2C). These genes include lineage-defining transcription factors (ASCL1, OLIG2, KLK6), organ- or tissue-specific markers (BGLAP, GATA4), and disease-relevant signaling and remodeling mediators (FGFR3, MMP9). Their recovery by GF_{CAB}, but not by Geneformer, supports the notion that our model captures biologically meaningful and context-specific signals beyond the baseline.

In addition, we performed a comparative analysis of the frequency distribution of low-frequency genes predicted by each model (Fig 2D). The results show that GF_{CAB} consistently predicts genes with lower prevalence compared to Geneformer, indicating a shift toward more diverse and less dominant gene predictions. This behavior aligns with our design objective of mitigating redundancy and promoting predictive diversity, which in turn facilitates the identification of rare but biologically informative genes.

Line number: 166-172.

Reviewer Comment 1.3 — The observation that larger-scale pretraining leads to worse zero-shot batch integration is interesting and deserves more discussion. While I understand that diagnosing the precise mechanism may be outside the scope of this study, including a short, dedicated discussion section on possible explanations would help contextualize this result.

Reply:

We thank the reviewer for this valuable suggestion. We agree that the observed degradation in zero-shot batch integration performance with increasing pretraining scale is an important finding that warrants further discussion.

Consistent with prior studies [1–3], foundation models such as Geneformer and scGPT [4] often underperform simpler baselines like scVI [5] in batch integration tasks. Notably, scVI is explicitly designed to model and disentangle batch effects through a probabilistic latent variable framework, whereas foundation models are primarily optimized for large-scale representation learning without an explicit batch-correction objective.

One possible explanation for our observation is that large-scale pretraining encourages the model to learn highly detailed and dataset-specific gene co-expression structures. While beneficial for capturing biological signal, these representations may also encode batch- or modality-specific biases present in the pretraining data. As the scale and diversity of the training data increase, the model may become more sensitive to such structured variations, which can hinder its ability to generalize across unseen batches in a zero-shot setting.

In contrast, models pretrained on smaller datasets are exposed to fewer such complex patterns and may therefore learn comparatively smoother or less specialized representations. This reduced specialization may incidentally improve their robustness to distributional shifts, resulting in better zero-shot batch integration performance.

We emphasize that this interpretation remains a hypothesis, as systematically disentangling the contributions of data scale, model capacity, and training dynamics is beyond the scope of the current study. Nonetheless, our findings highlight a potential trade-off between large-scale representation learning and cross-batch generalization, suggesting that additional mechanisms, such as explicit batch-invariance objectives or domain adaptation strategies, may be necessary to fully realize the benefits of large-scale pretraining in this context.

Line number: 214-222.

Reviewer Comment 1.4 — Both of the proposed modifications: cumulative-assignment suppres-

sion and similarity-based regularization; directly encourage the model to avoid repeated predictions across masked positions. Because repetition and uniqueness are also part of the evaluation metrics, it is difficult to determine how much of the reported improvement reflects better modeling versus optimization toward the metric itself. A discussion of this potential circularity, or an evaluation using alternative diversity measures, would make the claims stronger.

Reply:

We acknowledge that cumulative-assignment suppression and similarity-based regularization are explicitly designed to reduce redundant predictions, and therefore improvements in repetition- and uniqueness-based metrics alone could, in principle, reflect optimization toward these criteria rather than a genuine enhancement in modeling capacity.

To address this concern, we refer to the experiment discussed in Comment 1.1, where we evaluated a simple post hoc alternative that enforces uniqueness during inference without modifying the model itself. While this strategy completely eliminates repetition, it leads to a consistent decrease in masked token prediction accuracy, particularly for the baseline Geneformer model. This result demonstrates that improving diversity through naive constraints does not inherently preserve, and can even degrade, predictive performance.

In contrast, $\mathrm{GF_{CAB}}$ achieves both reduced redundancy and competitive (or improved) masked token prediction accuracy without requiring post hoc enforcement. This indicates that the model is not merely optimizing for diversity-related metrics, but is instead learning representations that better balance probability allocation across candidate genes. The improved robustness of $\mathrm{GF_{CAB}}$ under enforced-uniqueness conditions further supports this interpretation, suggesting that diversity is integrated into the learned structure of the model rather than imposed externally.

Additionally, our analysis of low-frequency gene recovery (Comment 1.2) shows that $\mathrm{GF_{CAB}}$ preferentially captures biologically meaningful but underrepresented genes that are often overlooked by the baseline. This provides an orthogonal line of evidence that the model's improvements extend beyond metric-specific optimization and reflect enhanced sensitivity to biologically relevant signals.

Taken together, these results suggest that the observed gains are not solely attributable to circular optimization of evaluation metrics, but rather arise from a more effective modeling of gene-level dependencies that improves both predictive diversity and overall representation quality.

Line number: 159-165.

Minor comments

Reviewer Comment 1.5 — Figure 1.3 (Fig 3 in the manuscript) is difficult to parse. The large number of tasks, models, and ranking metrics results in very dense panels. A redesign that separates task families or uses clearer labeling would help readers interpret the results more easily.

Reply:

We agree that the original version of Fig 3 was overly dense and could hinder interpretability due to the large number of tasks, models, and ranking metrics presented simultaneously.

In response, we have substantially redesigned the figure to improve clarity and readability. First, following the suggestion in Comment 2.9, we replaced rank-only representations with explicit numerical performance values for each downstream task (Fig 3A), allowing readers to directly assess model

performance without relying solely on relative rankings. To maintain interpretability, we retained color annotations to indicate relative ranking while adding clearer task and metric labels.

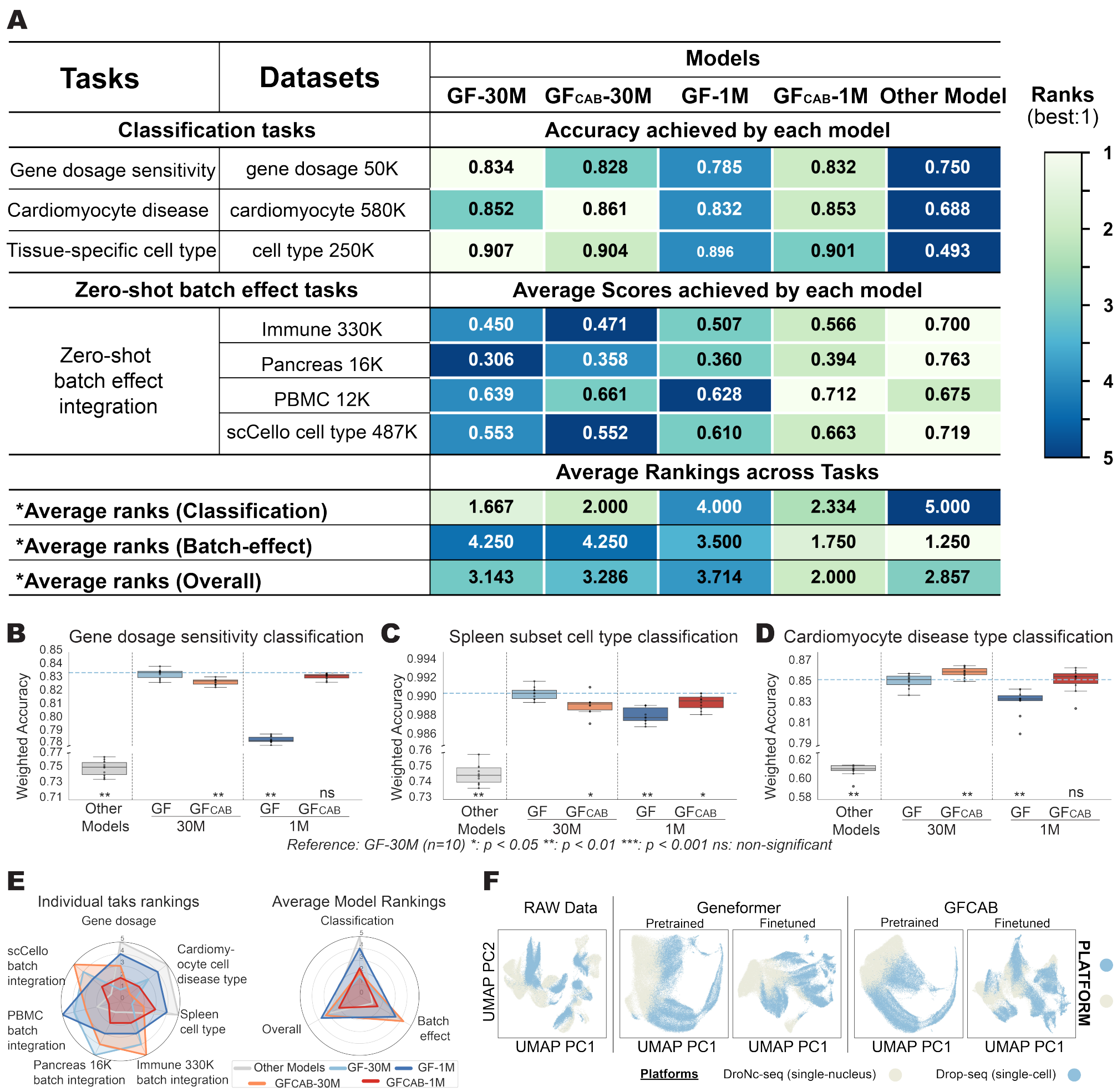

Tasks	Datasets	GF-30M	GFCAB-30M	GF-1M	GFCAB-1M	Other Model
**Classification tasks**		**Accuracy achieved by each model**				
Gene dosage sensitivity	gene dosage 50K	0.834	0.828	0.785	0.832	0.750
Cardiomyocyte disease	cardiomyocyte 580K	0.852	0.861	0.832	0.853	0.688
Tissue-specific cell type	cell type 250K	0.907	0.904	0.896	0.901	0.493
**Zero-shot batch effect tasks**		**Average Scores achieved by each model**				
Zero-shot batch effect integration	Immune 330K	0.450	0.471	0.507	0.566	0.700
	Pancreas 16K	0.306	0.358	0.360	0.394	0.763
	PBMC 12K	0.639	0.661	0.628	0.712	0.675
	scCello cell type 487K	0.553	0.552	0.610	0.663	0.719
		**Average Rankings across Tasks**				
***Average ranks (Classification)**		1.667	2.000	4.000	2.334	5.000
***Average ranks (Batch-effect)**		4.250	4.250	3.500	1.750	1.250
***Average ranks (Overall)**		3.143	3.286	3.714	2.000	2.857

Figure 3: Redesigned overview of model performance across downstream tasks, model architectures, and pretraining data scales. (A) Summary of model performance across three classification tasks (gene dosage sensitivity, cardiomyocyte disease-state classification, and spleen cell type classification) and four zero-shot batch integration benchmarks, with numerical metrics displayed alongside color-coded ranking indicators. (B–C) Detailed classification results for each task, showing comparisons between foundation models and the best-performing classical baselines (SVM, Random Forest, Logistic Regression). (E) Per-task ranking and average ranking across all tasks and datasets, with a radar plot illustrating comparative performance across models and data scales. (F) Visualization of embedding quality on cardiomyocyte datasets [6], including both Drop-seq (single-cell) and DroNc-seq (single-nucleus) modalities. Models are fine-tuned on single-cell data and evaluated in a zero-shot manner across modalities, demonstrating improved batch integration performance after fine-tuning.

Second, we reorganized the figure by grouping related tasks into coherent panels. Specifically, downstream classification results are separated and presented in dedicated panels (Fig 3B–C), where

only the best-performing baseline models (SVM, Random Forest, Logistic Regression) are shown for clarity. This reduces visual clutter and facilitates direct comparison between foundation models and strong baselines.

Third, for overall performance comparison, we retained average ranking as a summary metric but complemented it with a radar plot (Fig 3E), which provides a more intuitive visualization of model performance across tasks and pretraining scales.

Finally, we incorporated an additional evaluation on cardiomyocyte sequencing datasets [6], including both Drop-seq (single-cell) and DroNc-seq (single-nucleus) modalities (Fig 3F). In line with Comment 3.5, models were fine-tuned using single-cell data and evaluated in a zero-shot manner across both modalities. This addition further contextualizes model performance under biologically relevant distribution shifts and demonstrates improved batch integration following fine-tuning, consistent with prior observations [7].

Overall, these revisions aim to reduce visual complexity, improve logical organization, and enhance the interpretability of model comparisons across tasks and datasets.

Figure following paragraph: 180-190.

Reviewer Comment 1.6 — The introduction mixes broad method families (e.g., MLPs, DET) with specific models (e.g., GEARS, scANVI). Reorganizing this section to separate (i) general methodological families and (ii) representative examples would improve clarity.

Reply:

The introduction has been revised to improve clarity by explicitly separating general methodological families from representative models.

In particular, we reorganized the relevant section to first introduce broad categories of approaches for modeling single-cell transcriptomic data, including classical methods (*e.g.*, multilayer perceptrons and differential expression testing) and more recent deep generative and representation learning frameworks. We then present representative models within each category to illustrate their practical implementations and contributions. This restructuring provides a clearer conceptual progression from general modeling strategies to specific instantiations.

The revised text is as follows:

Characterizing genetic relationships within single-cell transcriptomic data [8, 9], and leveraging these representations for a broad spectrum of downstream biological tasks, has emerged as a central paradigm across modern computational biology and related disciplines [4, 10, 11]. To this end, a wide range of computational approaches has been developed to systematically learn latent genetic structures from such data and generalize these representations to predictive tasks, including perturbation response modeling, drug sensitivity prediction, disease classification, and cellular dynamics inference [12–15]. Early efforts adapted classical frameworks, such as multilayer perceptrons (MLPs) and differential expression testing (DET) [16], while subsequent work introduced specialized models tailored to single-cell data, including scVI, scANVI, and GEARS [5, 17, 18], collectively demonstrating the feasibility of extracting biological representations from sparse transcriptomic profiles. More recently, single-cell transcriptomic foundation models have been proposed as a unified representational framework, leveraging self-attention mechanisms [19] to capture complex gene–gene dependencies and enabling flexible adaptation to a wide range of downstream tasks through pretraining and fine-tuning. Emerging models such as scGPT, scFoundation, and SCmilarity [4, 20, 21] highlight the potential of this paradigm. In parallel, the Bidirectional Encoder Representations from Transformers (BERT) architecture [22] has been extended to single-cell

settings, as exemplified by the pioneering scBERT [23] and the subsequent Geneformer (GF), a ranking-based BERT variant pretrained on approximately 30 million cells [7], both of which demonstrate strong performance across a wide range of downstream tasks.

Line number: 2-23.

Conclusion from the reviewer: The manuscript supports its central conclusion that scale alone does not guarantee better downstream performance, within the scope of the presented evaluations. This aligns with emerging findings from other recent assessments of single-cell foundation models. Additional biological interpretation and deeper contextual discussion would further strengthen the contribution.

Reviewer 2

Summary from the reviewer:

This paper evaluates whether scaling pre-training data improves the performance of rank-based single-cell foundation models and introduces GFCAB, a modified Geneformer architecture aimed at increasing prediction diversity. Models trained on 1M versus 30M ranked single-cell profiles are assessed on masked-gene prediction, three classification tasks, and zero-shot batch-effect integration. Results show that larger datasets improve masked-token accuracy but do not consistently enhance downstream performance; smaller models sometimes generalize better. Rank-based approaches remain weak for batch correction compared to domain-specific tools. GFCAB improves predictive diversity and reduces reliance on large pre-training corpora but still trails specialized methods.

Major comments

Reviewer Comment 2.1 — Choice of baseline model is scientifically questionable

Geneformer (GF) is already well documented to underperform compared to more recent single-cell foundation models such as scGPT, scFoundation, and SCimilarity (Kedzierska et al., Genome Biology 2025). These methods incorporate numerical expression values rather than only rank-order information. The study's rationale is therefore weak unless the authors also include these stronger baselines. Evaluating GF and GF-CAB against these non-rank-based models under the same evaluation framework would provide essential context and greatly strengthen the study.

Reply:

We thank the reviewer for this important comment and agree that evaluating against stronger, non–rank-based foundation models provides essential context.

As noted, prior studies have demonstrated that Geneformer (GF) can underperform more recent models such as scGPT, scFoundation, and SCimilarity, particularly in tasks such as zero-shot batch integration [1–3, 24]. In the original manuscript, we focused on GF as the primary baseline because our work specifically targets limitations inherent to rank-based modeling, and GF represents the most widely adopted architecture in this category.

Following the reviewer's suggestion, we have extended our evaluation to include scGPT, scFoundation, SCimilarity, and scCello under a unified benchmarking framework. To ensure a fair comparison, we reprocessed all datasets from raw sequencing data to match the input requirements of each model, rather than relying on precomputed embeddings or external evaluation pipelines.

The expanded benchmark includes two classification tasks and zero-shot batch integration across four datasets. The classification tasks consist of (i) a multi-tissue classification task using eight tissues (bone marrow, eye, heart, kidney, large intestine, small intestine, liver, and muscle) from the Tabula Sapiens dataset [25], and (ii) a lung disease classification task (COPD, IPF, control) using dataset GSE136831 [26]. For batch integration, we evaluated models on Pancreas 16K [27], PBMC 12K [5, 28], Immune 330K [29], and the scCello OOD dataset (487K cells) [3]. Performance was assessed using consistent metrics across models, including accuracy and macro F1 for classification, and the combined AvgScore ($0.6\times$AvgBio $+ 0.4\times$AvgBatch) for batch integration.

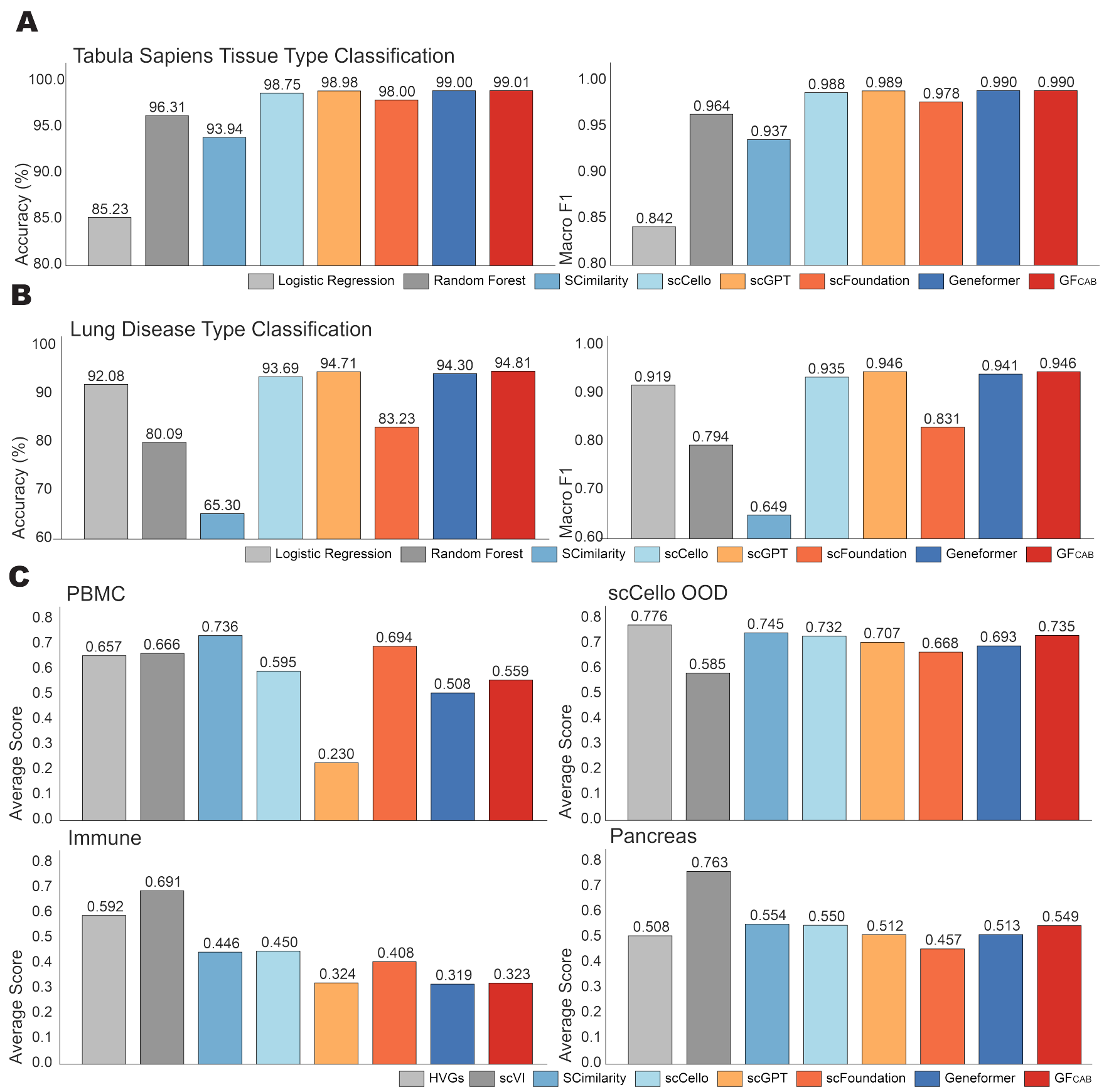

Figure 4: Benchmark comparison of rank-based and non–rank-based foundation models under a unified evaluation framework. (A) Tissue classification performance (accuracy and macro F1) across eight tissues from Tabula Sapiens. (B) Lung disease classification performance (COPD, IPF, control) evaluated using dataset GSE136831. (C) Zero-shot batch integration performance measured by AvgScore (0.6×AvgBio + 0.4×AvgBatch) across four datasets (Pancreas 16K, PBMC 12K, Immune 330K, and scCello OOD). Models include GF, GF_{CAB}, scGPT, scFoundation, SCimilarity, and scCello, with classical baselines (logistic regression and random forest) for classification and HVG-based representation and scVI for batch integration. GF_{CAB} consistently improves over GF while remaining competitive within the rank-based modeling paradigm.

The results are summarized in Fig 4. Overall, GF_{CAB} consistently improves upon GF across tasks. Importantly, we observe that GF_{CAB} closely tracks the performance trend of GF: in settings where GF performs competitively, GF_{CAB} further enhances performance; in settings where GF underperforms relative to non–rank-based models, GF_{CAB} yields modest improvements but does not fully close the gap.

These findings suggest that our proposed modifications effectively address limitations related to redundancy and diversity within the rank-based modeling paradigm, but do not fundamentally alter the representational constraints associated with rank-only inputs. We have added a clarification in the revised manuscript to explicitly acknowledge this limitation and to position GF_{CAB} as a complementary improvement within the Geneformer framework rather than a replacement for alternative modeling paradigms.

Line number: 274-292.

Reviewer Comment 2.2 — Incorrect claim regarding the first transformer-based single-cell model

The manuscript states that GF was the first BERT-style model for single-cell transcriptomics. This is factually incorrect. scBERT preceded Geneformer and should be acknowledged appropriately.

Reply:

We thank the reviewer for pointing out this inaccuracy. We acknowledge that scBERT was the first study to apply a BERT-based architecture to single-cell transcriptomic data, and this should be properly recognized.

In the original manuscript, our intention was to highlight Geneformer as the first model to adapt a BERT-style architecture specifically to rank-ordered single-cell transcriptomic inputs with biologically motivated constraints. However, we agree that the wording is ambiguous and may be interpreted as a broader claim regarding the first application of transformer models in this domain.

To address this, we have revised the manuscript to explicitly distinguish these contributions. We now correctly attribute scBERT as the first application of BERT to single-cell RNA-seq data, and clarify that Geneformer represents a subsequent development that introduces a rank-based encoding scheme tailored to transcriptomic data.

Line number: 18-23.

Reviewer Comment 2.3 — Missing comparison to scCello, a key rank-based foundation model

Another rank-based model, scCello, uses cell ontology to guide pre-training. The authors use scCello's cell-type data but do not compare against the scCello model itself. This comparison is essential to determine whether GFCAB offers improvements over other augmented rank-based methods beyond GF alone.

Reply:

This is an important suggestion and we agree that scCello represents a relevant comparison point as an augmented rank-based foundation model that incorporates cell ontology information during pre-training.

In the original manuscript, we focused on comparisons within purely rank-based architectures and therefore did not include scCello, as its use of ontology-guided supervision introduces additional external information beyond rank-only inputs. While this design difference complicates direct attribution of performance gains, we agree that including scCello is essential for contextualizing the effectiveness of GF_{CAB} relative to other extensions of the Geneformer framework.

Following the reviewer's suggestion, we have incorporated scCello into our unified benchmarking framework alongside GF, GF_{CAB}, and other foundation models (Fig 4). All models were evaluated under consistent data preprocessing and evaluation protocols.

The results show that in tasks where rank-based models perform competitively, scCello does not consistently outperform GF, whereas GF_{CAB} provides more reliable improvements over the baseline. In contrast, in more challenging settings, particularly those where rank-based models underperform, scCello benefits from the incorporation of ontology-guided structure and can achieve stronger performance than

both GF and GF_{CAB}. However, this advantage is not uniform across tasks, and scCello does not consistently outperform GF_{CAB} in all evaluated scenarios.

These findings suggest that GF_{CAB} improves the intrinsic modeling capacity of rank-based architectures without relying on external ontological priors, while scCello leverages additional structured supervision to enhance performance in specific settings. We now acknowledge this in the revised manuscript and clarify this distinction positioning GF_{CAB} as a complementary approach within the rank-based modeling framework.

Line number: 274-292.

Reviewer Comment 2.4 — Lack of explanation for greater diversity in smaller-scale models

The manuscript claims that smaller-scale models exhibit greater exploratory diversity, but offers no mechanistic or empirical explanation. This is a central observation and should be justified biologically, algorithmically, or statistically.

Reply:

We thank the reviewer for highlighting the need for a clearer explanation of this observation. We agree that the increased predictive diversity observed in smaller-scale models warrants further clarification.

From an algorithmic and statistical perspective, one plausible explanation is that models trained on smaller-scale datasets are exposed to fewer and less complex co-expression patterns, resulting in less specialized or over-constrained representations. In contrast, large-scale pretraining encourages the model to learn highly consistent and dominant gene–gene relationships present across the dataset. While this improves overall likelihood-based prediction, it can also lead to sharper probability distributions concentrated on a limited set of high-frequency genes, thereby reducing exploratory diversity during masked prediction.

Smaller-scale models, by comparison, tend to learn smoother or less peaked predictive distributions due to reduced exposure to such dominant patterns. This effectively increases the entropy of the model's output distribution, allowing a broader set of candidate genes, including lower-frequency ones, to be selected during prediction. This behavior is consistent with our empirical observations that smaller-scale models exhibit higher uniqueness and lower repetition.

We further note that similar trends have been observed in prior work and in our own experiments, where simpler or less specialized models (*e.g.*, scVI and SCimilarity) often demonstrate greater exploratory behavior compared to large-scale foundation models. Additionally, as discussed in Comment 3.3, extending training on smaller datasets can partially close the performance gap while still preserving higher diversity, suggesting that both data scale and training dynamics contribute to this effect.

We emphasize that this explanation remains a hypothesis based on observed empirical trends, and a systematic disentanglement of data scale, model capacity, and training duration is beyond the scope of the current study. We have added a dedicated discussion in the revised manuscript to clarify these potential mechanisms and to better contextualize this observation.

Line number: 123-158.

Reviewer Comment 2.5 — Missing specification of "Other Models" in Fig 3A

Figure 3A reports rankings among "Other Models," but the manuscript does not specify what those models are. For meaningful interpretation, the figure should explicitly list scVI, Harmony, or other clustering/integration tools, as well as supervised baselines (e.g., SVM, logistic regression).

Reply:

We agree with reviewer 2 that the term "Other Models" in Fig 3A (now Fig 3) was insufficiently specified and could hinder proper interpretation of the results.

To address this issue, we have revised both the figure caption to explicitly list the baseline models included in each task category, rather than only mentioning these methods in the *Methods* section. Specifically, for the downstream classification tasks, we include logistic regression, random forest, and support vector machine (SVM) as supervised baselines. For clarity and readability, we report the performance of the best-performing baseline model for each task in the figure.

For the zero-shot batch integration tasks, we include highly variable gene (HVG)-based representations and scVI as baseline methods. Similarly, we present the best-performing baseline among these methods for each dataset.

These revisions ensure that all compared methods are clearly defined, improving the transparency and interpretability of the figure.

Line number: 180-190.

Reviewer Comment 2.6 — The fact that GFCAB-30M underperforms GF-30M needs more explanation

The observation that GFCAB-30M performs worse than GF-30M in certain tasks (Fig 3B–C) contradicts the stated motivation for model refinements. The manuscript must explain this behavior—whether due to over-regularization, suboptimal hyperparameters, or model instability.

Reply:

We believe that this trade-off, namely the underperformance of GF_{CAB}-30M compared to GF-30M, may originate from overfitting which could be beneficial to the model in tasks in which downstream data are derived from the pretraining corpus.

Our proposed modifications, cumulative-assignment suppression and similarity-based regularization, introduce an explicit regularization effect by discouraging redundant predictions and flattening overly concentrated probability distributions across masked positions. While this improves predictive diversity and can enhance generalization, it may also reduce the model's ability to fully exploit highly specific patterns present in the pretraining data.

Notably, the two tasks where GF_{CAB}-30M underperforms GF-30M (Fig 3B–C) are spleen-specific cell type classification and gene dosage sensitivity classification. Both tasks are derived directly from subsets of the Genecorpus-30M pretraining dataset, with consistent preprocessing and only additional label annotations. In this setting, GF-30M can benefit from stronger memorization of dataset-specific structures, effectively leading to better in-distribution performance.

In contrast, GF_{CAB}-30M, due to its regularization, is less prone to overfitting these dataset-specific patterns and therefore may exhibit slightly reduced performance on such in-distribution tasks. This interpretation is consistent with a classical bias–variance trade-off, where increased regularization can reduce overfitting at the expense of peak performance on data closely aligned with the training distribution.

Importantly, this trend reverses when evaluating on out-of-distribution or independently processed datasets. For example, in the cardiomyocyte disease classification task (Fig 3D), which uses an external

dataset processed independently of Genecorpus-30M, GF_{CAB}-30M outperforms GF-30M. A similar pattern is observed in the extended benchmarking results (Fig 4), where datasets are reprocessed outside the original pretraining pipeline. In these settings, GF_{CAB}-30M consistently matches or exceeds the performance of GF-30M.

Taken together, these results suggest that the observed underperformance is not due to model instability or suboptimal optimization, but rather reflects a trade-off introduced by regularization: GF_{CAB} sacrifices a degree of in-distribution fitting in order to achieve improved generalization and predictive diversity. We have clarified this point in the revised manuscript to better contextualize the behavior of the model across different evaluation settings.

Line number: 191-213.

Reviewer Comment 2.7 — Study examines data scaling but not model scaling

The authors explore the effect of data scale (1M vs. 30M profiles) but not model scale. Scaling laws in transformers often require increased parameter counts—e.g., deeper networks, larger embedding dimensions—for benefits from larger datasets to manifest. The authors should discuss (or ideally test) whether GF or GFCAB benefits more from increased model capacity at larger data scales.

Reply:

We agree that model scaling is an important factor in transformer-based architectures and that understanding its interaction with data scale is essential.

To address this, we conducted additional experiments to systematically evaluate the effect of model capacity. Specifically, we pretrained both GF and GF_{CAB} models on the full Genecorpus-30M dataset while varying architectural parameters, including the number of transformer layers, embedding dimensions, and attention heads. For consistency, we report model scale in terms of the total number of trainable parameters (in millions), spanning a range from 4.35M to 26.97M parameters.

All models were evaluated under a unified framework, including masked token prediction during pretraining and two downstream classification tasks (tissue-type classification and lung disease classification), as described in Comment 2.1. The results are summarized in Fig 5.

We observe that increasing model capacity initially leads to improved performance across both pretraining and downstream tasks, consistent with expected scaling behavior in transformer models. However, beyond a certain scale, performance begins to plateau or degrade, suggesting that the available training data may be insufficient to fully support larger model capacities. This behavior aligns with known scaling dynamics, where optimal performance depends on a balance between model size and data scale.

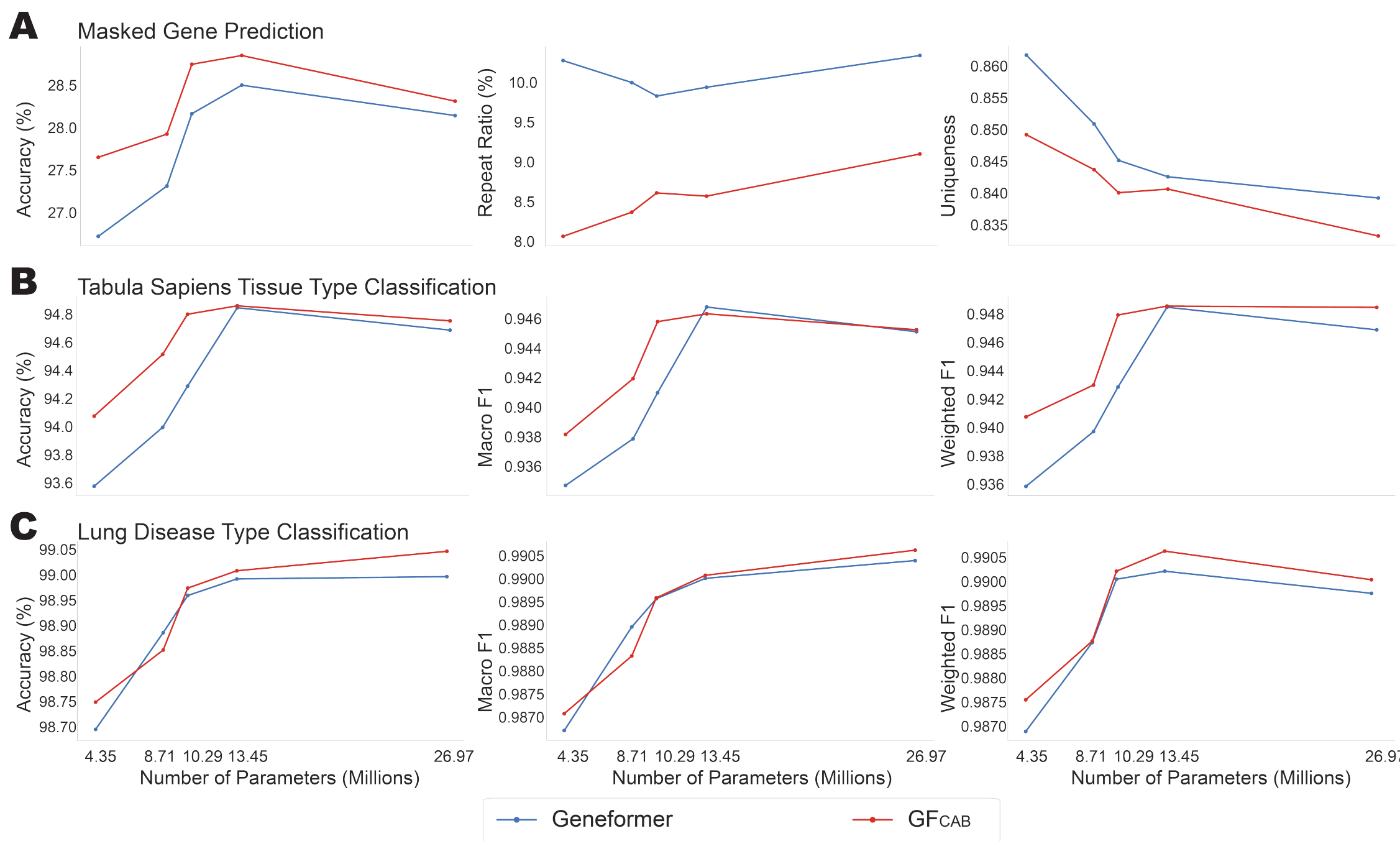

Figure 5: Effect of model scaling on performance for GF and GF_{CAB}. Model scale is measured by the number of trainable parameters (ranging from 4.35M to 26.97M). (A) Pretraining performance, including masked token prediction accuracy, repetition ratio, and uniqueness. (B) Tissue-type classification performance (accuracy, macro F1, weighted F1). (C) Lung disease classification performance (COPD, IPF, control; accuracy, macro F1, weighted F1). Moderate increases in model size improve performance, while excessive scaling leads to diminishing or negative returns, likely due to data–model mismatch. GF_{CAB} consistently outperforms GF across all model scales.

Importantly, across all model scales, GF_{CAB} consistently outperforms GF in terms of masked token prediction accuracy and downstream classification performance, while also maintaining lower repetition ratios. This indicates that the benefits of our proposed modifications are complementary to model scaling and persist across different capacity regimes.

We have added a discussion in the revised manuscript to highlight these findings and to emphasize that both data scale and model capacity jointly influence performance, suggesting that future work could further explore optimal scaling strategies for rank-based single-cell foundation models.

Line number: 147-151.

Minor comments

Reviewer Comment 2.8 — Figure 1 font size too small

The text and labels in Fig 1 are difficult to read and should be enlarged.

Reply:

We appreciate the reviewer for pointing out that the original version of Fig 1 suffered from limited readability due to small font sizes and dense visual elements.

In response, we have redesigned the figure to improve clarity and presentation. Specifically, we increased font sizes across all labels and annotations, simplified text content to reduce visual clutter, and reorganized the layout to better separate conceptual components. We also refined the color scheme and graphical elements to enhance contrast and readability. These changes ensure that the pretraining workflow, downstream evaluation tasks, and the GF_{CAB} architecture are more clearly conveyed.

The revised figure is provided below (Fig 6).

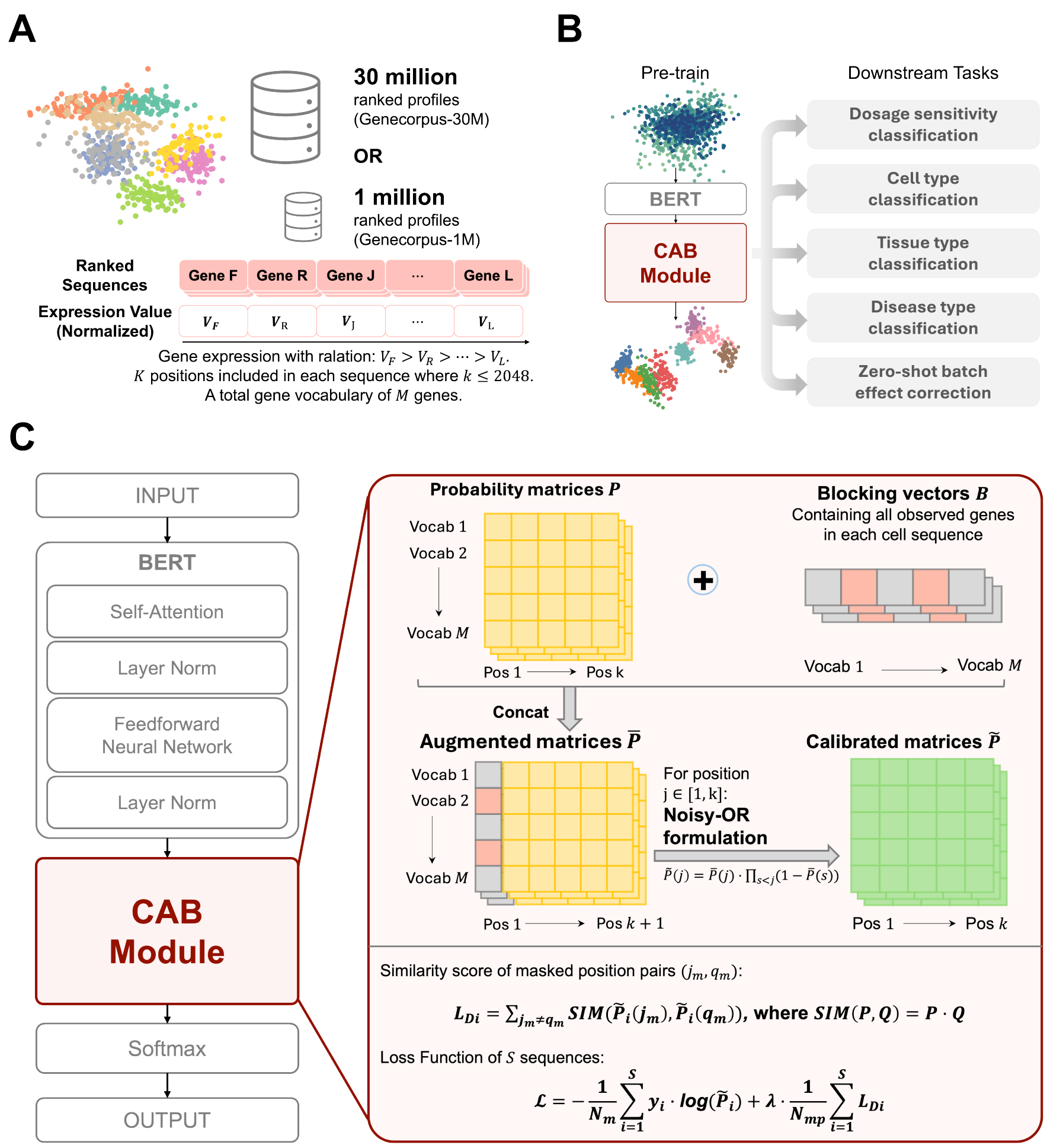

Figure 6: Redesigned schematic overview of the modeling framework and evaluation pipeline. (A) Pretraining is performed on two data scales, 1 million (1M) and 30 million (30M) single-cell profiles, where each cell is represented as a rank-ordered gene sequence based on expression levels, with a vocabulary exceeding 20,000 genes. (B) Pretrained models are evaluated across multiple downstream tasks, including gene dosage sensitivity classification, cell type classification, tissue type classification, disease type classification, and zero-shot batch integration. (C) Illustration of the GF_{CAB} architecture, highlighting the cumulative-assignment suppression and similarity-based regularization components introduced during pretraining to reduce repetition and enhance predictive diversity.

Figure following paragraph: 41-55.

Reviewer Comment 2.9 — Present numerical values instead of rankings in Fig 3A

It would be more informative to include raw numerical performance metrics rather than a ranking-only figure. Best-performing methods should be bolded. Typically, this information is conveyed via a table rather than a figure.

Reply:

We agree that presenting numerical values improves interpretability compared to ranking-only visualizations.

In response, we have redesigned Fig 3 (see also Comment 1.5) to explicitly report raw numerical performance metrics for each model across all downstream tasks (Fig 3A). This enables direct and transparent comparison without relying solely on relative rankings. To preserve an overview of relative performance and trends across models and tasks, we retain color annotations to indicate ranking alongside the numerical values, supplemented with clearer task and metric labels.

In addition, ranking results are still included in Fig 3A to provide a concise summary of relative performance across classification tasks, zero-shot batch integration tasks, and overall model performance.

Overall, these revisions ensure that the figure provides both quantitative detail and an intuitive visual summary, addressing the reviewer's concern.

Figure following paragraph: 180-190.

Reviewer 3

Summary from the reviewer:

The authors propose an improvement to Geneformer to limit repetition of genes and similarity of predictions for masked learning and test the performance of this method vs. the original Geneformer with 1 or 30M cell pretraining datasets on several downstream tasks, also comparing to baselines. The authors test both biologically-relevant downstream tasks (gene dosage sensitivity, cardiomyocyte cell type classification, and tissue-specific cell type classification) as well as the preprocessing task of batch correction.

The study is a useful comparison of different dataset sizes and an interesting architectural modification to improve the pretraining. However, the overarching conclusions of the paper as written seem to focus almost entirely on the batch effect results and ignore the biologically-relevant task results. At the end of the day, the biologically relevant tasks are what biologists would like to study, and the batch effect correction is simply a preprocessing means to an end. One finding in the data of the paper that is not discussed by the authors is that it appears that batch effect integration is correlated with worse performance on biologically relevant tasks. This raises the question of why the field is so focused on batch effect integration if it actually leads to worse performance on the tasks relevant to the true biological questions at hand. Overall, the text should be revised to not cherry pick the results to overweight the batch effect correction, and should instead objectively report the findings.

Major comments

Reviewer Comment 3.1 — Fig 3 indicates that the Geneformer variants significantly outperformed other models in all of the biologically relevant tasks. If there are lower batch effects but the models do worse in distinguishing biologically relevant features, then it seems that batch effect integration does not need to be optimized for these tasks and in fact correcting the batch effects may be removing true biological signal that is necessary for the biologically relevant tasks. It is unclear why the authors would equally weight the 1 task of batch correction on 3 datasets with the 3 distinct biological tasks (which are folded into 1 for the averaging). Since batch effects are a preprocessing task for the purpose of improving biological task performance, it would seem the biological tasks should take priority, rather than reducing them to a single point. This cherry-picking of the results to overweight batch correction in the discussion leads to the conclusions of the paper not being well-supported by the results.

Reply:

The reviewer raises an important concern regarding the relative weighting of biological tasks versus batch integration, and the potential risk of overemphasizing batch correction in interpreting model performance.

The primary objective of this study is twofold: (i) to improve pretraining alignment, specifically by reducing repetitive predictions and enhancing predictive diversity, while maintaining competitive performance on biologically meaningful downstream tasks; and (ii) to evaluate whether these improvements translate into better generalization across datasets, as assessed by zero-shot batch integration.

Biologically relevant downstream tasks remain the primary benchmark for model utility. As shown in Fig 3A–D, Geneformer (GF) variants, including GF_{CAB}, consistently perform strongly on classification tasks, indicating that the proposed modifications do not compromise the model's ability to capture biologically meaningful signals. Furthermore, in benchmarking against other foundation models (Fig 4), GF_{CAB} consistently improves upon Geneformer across both classification and batch integration tasks. Importantly, we do not observe a systematic trade-off in which improved batch integration leads to degraded biological task performance.

Table 1: Ranking results across three classification tasks and zero-shot batch integration evaluated on four datasets. Overall rankings are computed using two strategies: (i) treating each batch integration dataset as an independent task; and (ii) aggregating all batch integration results into a single task before averaging with the three classification tasks. Across both strategies, the smaller-scale pretrained GF_{CAB} model achieves the strongest overall performance, supporting the conclusion that a reduced pretraining scale combined with the proposed architectural modifications can maintain competitive performance while improving generalization to unseen datasets.

Task / Dataset	GF-30M	GF_{CAB}-30M	GF-1M	GF_{CAB}-1M	Other
**Classification Tasks**					
Gene dosage sensitivity	1	3	4	2	5
Cardiomyocyte classification	3	1	4	2	5
Tissue-specific classification	1	2	4	3	5
Avg. rank (Classification)	1.667	2.000	4.000	2.334	5.000
**Zero-shot Batch Effect**					
Immune (330K)	4	5	3	2	1
Pancreas (16K)	5	4	3	2	1
PBMC (12K)	4	3	5	1	2
scCello OOD (487K)	4	5	3	2	1
Avg. rank (Batch)	4.250	4.250	3.500	1.750	1.250
**Overall Ranking Strategies**					
**Separate batch tasks**	3.143	3.286	3.714	**2.000**	2.857
**Integrated batch tasks**	2.313	2.563	3.875	**2.188**	4.063

Current research often uses batch effect correction performance as a proxy for assessing a model's representational capacity on large-scale pretraining corpora, with the broader goal of developing models that generalize effectively across datasets and can be transferred to new tasks without retraining.

We agree that biologically relevant downstream tasks should remain the primary focus, as these directly reflect the scientific questions of interest. At the same time, we consider zero-shot batch integration to be an important complementary evaluation, as it provides a practical measure of cross-dataset generalization. Given the substantial heterogeneity across single-cell datasets, arising from differences in experimental protocols, platforms, and biological contexts. Robust generalization is essential for enabling the reuse of pretrained models on new data. Once a model demonstrates compatibility with unseen datasets, it becomes feasible to apply it to downstream biological analyses within those datasets without additional retraining.

Accordingly, we include zero-shot batch integration not as a primary objective, but as a proxy for

generalization capability. We acknowledge, however, that treating multiple batch integration datasets equivalently to distinct biological tasks may introduce bias in aggregated rankings, and we explicitly address this in our revised evaluation.

To address this, we report two alternative ranking strategies (Table 1): (i) treating each batch integration dataset as an independent task, and (ii) aggregating all batch integration results into a single task before combining with classification tasks. Across both strategies, the relative foundation model performance trends remain consistent, and the smaller-scale GF_{CAB} model continues to demonstrate competitive overall performance.

The manuscript has been revised to clarify that batch integration is used to assess generalization rather than to outweigh biological task performance, and to explicitly present both ranking strategies to mitigate potential bias. This ensures that the conclusions are supported by a balanced evaluation across biologically relevant and generalization-oriented tasks.

Line number: 223-229.

Reviewer Comment 3.2 — Scaling is not only about increasing data but also increasing model size accordingly. There is generally a number of parameters that are ideal for a given amount of data, and so adding more data without increasing the size of the model would have no benefit, whereas increasing the size of the model would indeed show a benefit. The authors should test this and should also report the parameter size of the model, as this was not readily noted in the text.

Reply:

As noted in Comment 2.7, the relationship between model capacity and data scale plays a central role in the performance of transformer-based architectures.

To further investigate this aspect, we performed additional experiments examining the impact of model size. Both GF and GF_{CAB} were pretrained on the full Genecorpus-30M dataset while systematically varying architectural configurations, including depth and embedding dimensions. Model capacity is quantified by the total number of trainable parameters (in millions), ranging from 4.35M to 26.97M. We now explicitly report parameter counts for all model variants in the revised manuscript.

All models were evaluated within a consistent framework, including masked token prediction during pretraining and two downstream classification tasks (tissue-type and lung disease classification), as described in Comment 2.1. The corresponding results are presented in Fig 5.

Our findings comply with reviewer's statement that increasing model size yields performance gains up to a certain point, after which improvements diminish or reverse. This behavior suggests that larger models may not be fully supported by the available training data, leading to suboptimal utilization of capacity. These observations reinforce the reviewer's point that effective scaling requires coordination between model size and data availability, rather than increasing either dimension in isolation.

Across all tested configurations, GF_{CAB} consistently surpasses GF in both pretraining accuracy and downstream classification performance, while also reducing prediction redundancy. This indicates that the proposed modifications provide benefits that are robust to changes in model scale.

The revised manuscript now includes explicit reporting of model parameter sizes and an expanded discussion on the interplay between data scale and model capacity, emphasizing the importance of balanced scaling strategies in this setting.

Line number: 147-151.

Reviewer Comment 3.3 — Since the dataset 1M is simply a subsample of 30M and represents the same diversity, the paper seems to analyze the net benefit of training with the rest of the 29M cells. However, the 1M subsample benefits from the diversity of the data curation efforts for 30M cells, so is not really indicative of training with only a 1M cell corpus. The authors should revise the text to make this clear. If one imagines a loss curve, most of the gains are had in the beginning portion on the compute optimal frontier, so the authors are likely essentially testing the difference between stopping the training early or continuing for the remainder of the 30M cells.

Reply:

This is an important point regarding the interpretation of the 1M subset and its relationship to the full 30M dataset. We agree that the 1M data is not an independently curated corpus, but rather a subsample of Genecorpus-30M, and therefore benefits from the same underlying data diversity and curation process. As such, our original comparison may partially reflect differences in training dynamics (*e.g.*, training duration) rather than purely the effect of additional data.

To better disentangle these factors, we performed additional experiments comparing (i) early stopping during pretraining on the full 30M dataset and (ii) extended training on the 1M subset. Specifically, taking the total number of training steps used for 30M pretraining as a reference, we trained GF_{CAB} models at multiple fractions of this budget (5%, 10%, 20%, 30%, 50%, and 80%) under both data scales. In addition, we trained both GF_{CAB}-30M and GF_{CAB}-1M using an equivalent number of training steps corresponding to the 1M setting, to enable a more controlled comparison. The results are shown in Fig 7.

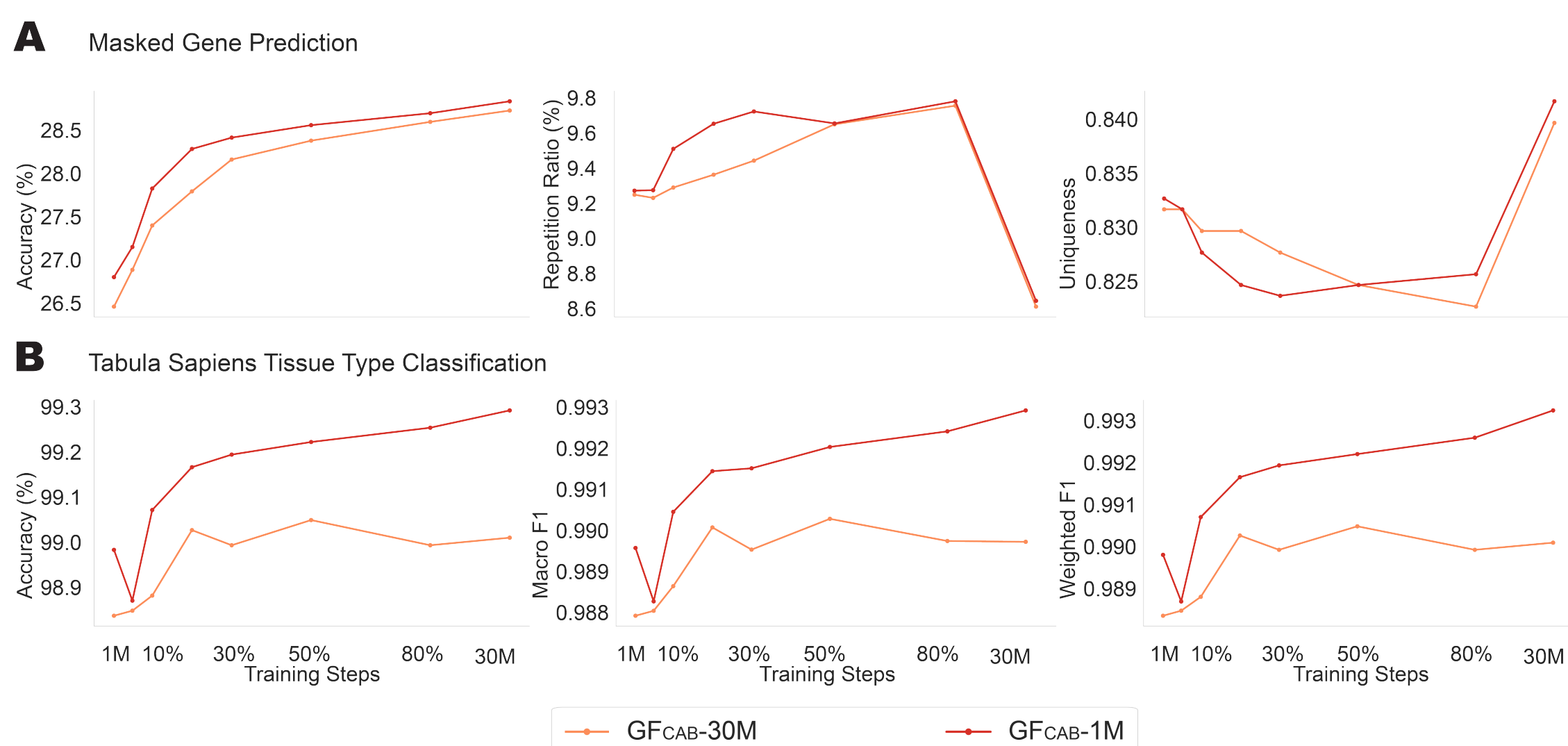

Figure 7: Comparison between elongating training on 1M data and early stopping on 30M data to investigate the net effect brought by the remaining 29M data. (A) Pretraining masked token prediction accuracy, repetition ratios, and uniqueness across different data scales and different training steps. (B) Tissue type classification accuracy, macro F1 score, and weighted F1 score across data scales and different training steps.

We observe that a substantial portion of performance gains, both in masked token prediction and downstream classification, occurs in the early stages of training, consistent with the reviewer's intuition. Moreover, models trained on the 1M subset with extended training can achieve performance comparable

to, and in some cases exceeding, models trained on the full 30M dataset with same effective training steps. These results suggest that training duration and optimization dynamics play a significant role alongside dataset size.

Importantly, we do not interpret these findings as evidence that the additional 29M cells are unnecessary. Rather, they indicate that the marginal benefit of scaling data may diminish under fixed model capacity and training regimes, and that a substantial portion of the learnable signal is captured early in training. We have revised the manuscript to clarify that the 1M subset inherits diversity from the full dataset, and that our analysis primarily reflects the interplay between data scale and training dynamics, rather than a pure comparison of independently curated datasets.

Line number: 138-158.

Reviewer Comment 3.4 — The authors should add baselines to the pretraining accuracy such as the naive method of selecting the most common gene for that position. For example, if Gene A was the most commonly located within the pretraining corpus at position 3, then the naive guess for position 3 would be Gene A. (The top likely gene should be calculated from the pretraining corpus and not the held out data on which the prediction is being performed.)

Reply:

Incorporating a naive baseline for pretraining evaluation is a helpful and well-motivated suggestion, particularly in the context of masked token prediction on rank-ordered sequences, where standardized reference baselines are not yet well established. Introducing such a baseline provides an intuitive point of comparison and helps contextualize the extent to which the observed performance reflects meaningful modeling capacity beyond simple frequency-driven patterns.

To this end, we implemented a simple frequency-based baseline. For each of the 2,048 positions, we computed the most frequently occurring gene in the pretraining corpus and used it as the default prediction for that position. Importantly, these frequencies were derived exclusively from the pretraining data, ensuring no information leakage from the evaluation set. Predictions were then generated for the same masked positions used in model evaluation, allowing direct comparison across metrics.

The results are summarized in Fig 8. This naive baseline achieves less than 2% accuracy on masked token prediction, while exhibiting a high repetition ratio (exceeding 80%), reflecting a strong bias toward frequently occurring genes. This behavior indicates that position-wise frequency alone is insufficient to capture the structured dependencies present in rank-ordered gene sequences, and tends to overlook less frequent but biologically informative genes.

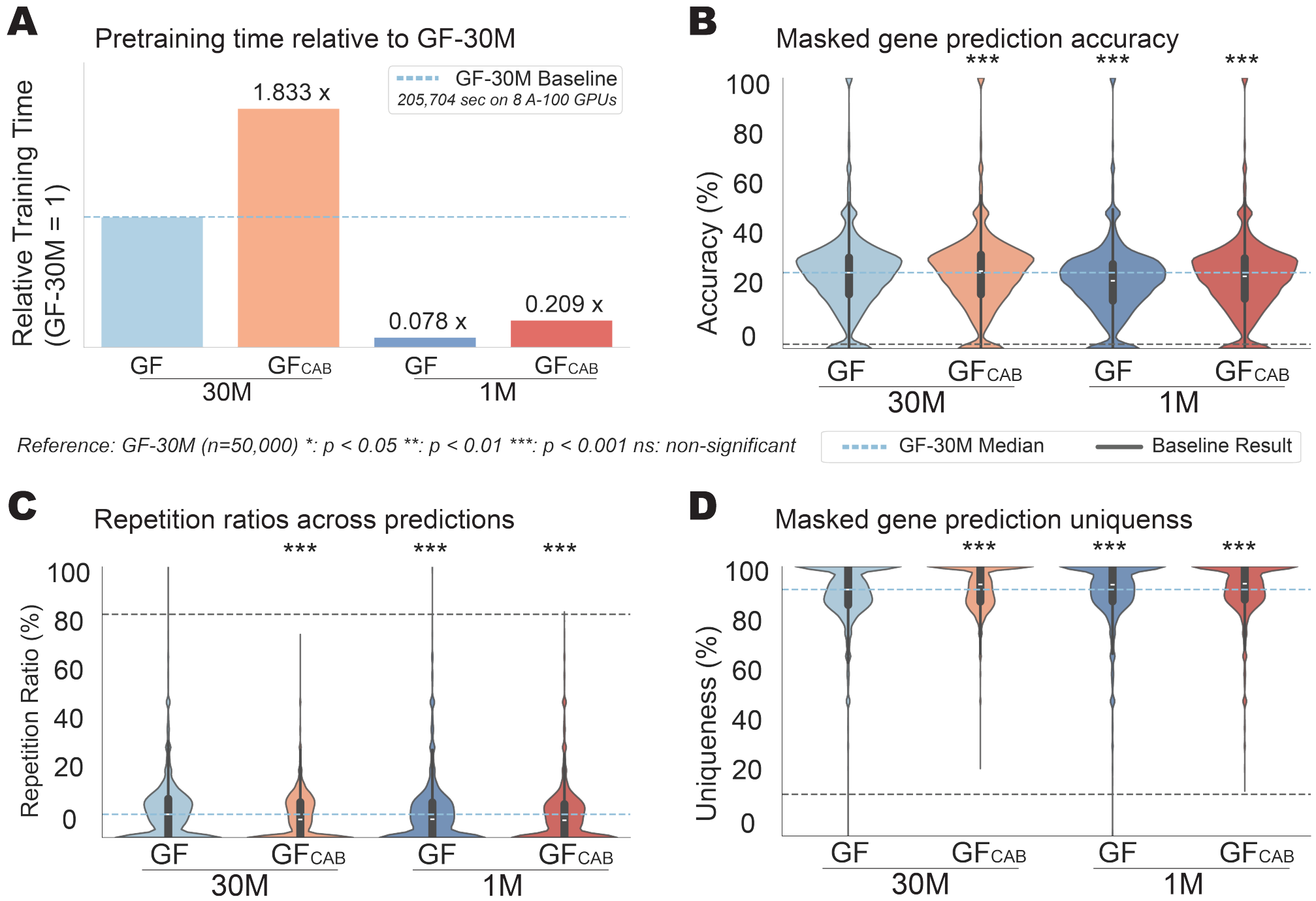

Figure 8: Pretraining metrics with baseline reference added. (A) Models were pretrained on 8 A100 GPUs under the same settings, and relative training times were computed with respect to GF trained on 30M cells (GF-30M). (B) Distributions of pretraining accuracy across GF and GF_{CAB} models at the 30M and 1M data scales; Wilcoxon tests were performed against GF-30M, and white lines indicated the median of each violin plot. (C) Distributions of overall repetition ratios across GF and GF_{CAB} models at both scales; Wilcoxon tests were conducted against GF-30M. (D) Distributions of uniqueness scores across GF and GF_{CAB} models at both scales; Wilcoxon tests were conducted against GF-30M. Baseline results for the masked token accuracy, repetition ratios, and uniqueness are indicated by dashed gray lines in B-D.

In contrast, both GF and GF_{CAB} substantially outperform this baseline across all metrics. Notably, GF_{CAB} further improves upon GF by achieving higher prediction accuracy while reducing repetition and increasing uniqueness. These results demonstrate that the observed performance gains are not attributable to simple frequency effects, but instead reflect the models' ability to learn meaningful gene–gene relationships.

Line number: 113-122.

Reviewer Comment 3.5 — The original paper indicated that batch effects are improved by fine-tuning on biologically relevant labels. Since other tools are explicitly designed for batch correction, the authors should test Geneformer with the suggested batch effect correction method. For example, the original paper performed cell type fine-tuning on single-cell RNAseq data and tested the batch integration of single-nucleus RNAseq data without further training.

Reply:

This is a valuable suggestion that highlights an important aspect of evaluating batch integration in the context of biologically informed fine-tuning. We agree that assessing whether fine-tuning on biologically relevant labels improves cross-modality integration is essential, particularly given prior observations in the original Geneformer study [7]. Incorporating such an experiment provides a more realistic evaluation scenario, where pretrained models are adapted to specific biological contexts and subsequently applied to related but distinct data modalities.

To investigate this, we followed a similar experimental design to the original Geneformer work using the dual-modality cardiomyocyte sequencing dataset [6]. In this dataset, batch labels correspond to sequencing platforms, namely Drop-seq (single-cell RNA-seq) and DroNc-seq (single-nucleus RNA-seq). Both GF and GF_{CAB} models were fine-tuned on the single-cell RNA-seq data using a supervised classification objective. The fine-tuned models were then used to generate embeddings for the combined dataset containing both single-cell and single-nucleus data, enabling evaluation of zero-shot batch integration without additional training on the single-nucleus modality.

The results are shown in Fig 9. We compare embeddings derived from highly variable gene (HVG)-based representations, pretrained GF and GF_{CAB} models, and their fine-tuned counterparts. As expected, pretrained models exhibit clear separation between sequencing modalities, indicating the presence of batch effects. After fine-tuning on biologically relevant single-cell data, both GF and GF_{CAB} demonstrate substantially improved integration between single-cell and single-nucleus modalities, consistent with previous findings [7].

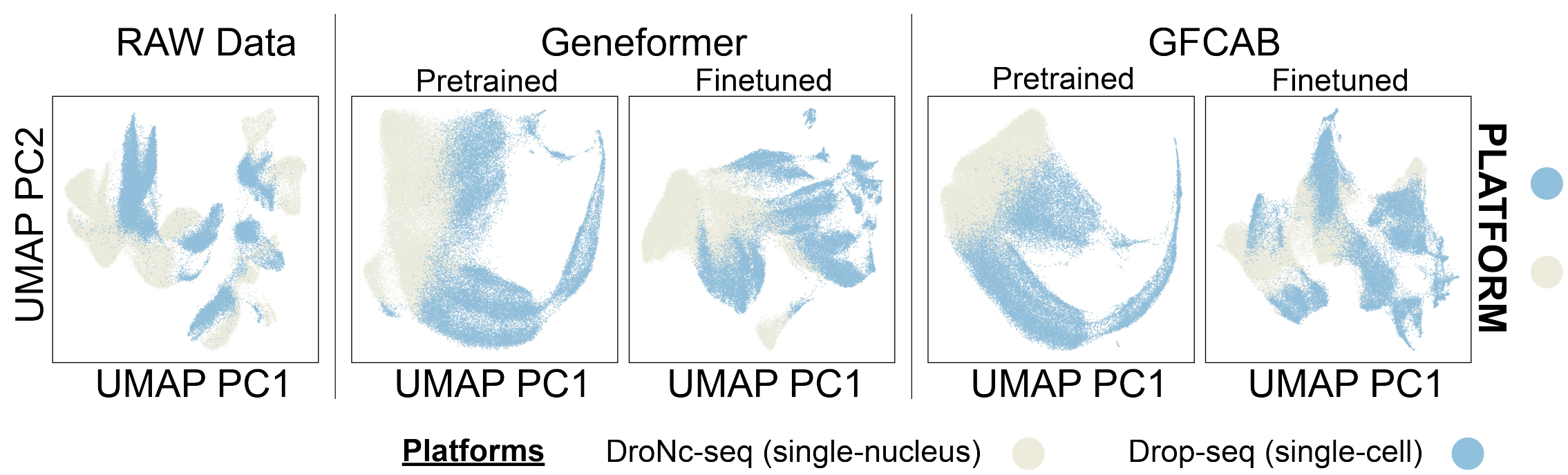

Figure 9: UMAP visualization of cell embeddings colored by sequencing platform (Drop-seq and DroNc-seq) for different representation methods. Shown are embeddings derived from HVG-based raw data, pretrained GF and GF_{CAB} models, and models fine-tuned on single-cell and single-nuleus RNA-seq data. Pretrained models exhibit clear separation between modalities, indicating batch effects. Fine-tuning on single-cell data substantially improves integration of single-nucleus data without additional training, with GF_{CAB} demonstrating enhanced integration compared to GF.

Notably, GF_{CAB} shows further improvement over GF after fine-tuning, with more effective mixing of cells across modalities. This suggests that the proposed architectural modifications not only enhance pretraining behavior but also improve the model's adaptability during fine-tuning, leading to stronger cross-modality generalization.

Line number: 230-235.

Reviewer Comment 3.6 — Currently the architectural modifications proposed by the authors

are not sufficiently discussed in the main text of the paper. The method should be further outlined in the main text so readers have an intuitive understanding of the modifications.

Reply:

The architectural modifications were previously described primarily in the *Methods* section, which may have limited their accessibility to readers seeking an intuitive understanding from the main text.

In the revised manuscript, we have added a dedicated subsection titled *"GF_{CAB} is a model architecture aligned with rank-ordered single-cell transcriptomic profiles"* within the *Results* section. This addition provides a clearer, high-level explanation of the proposed cumulative-assignment suppression and similarity-based regularization components, along with their intended effects on model behavior.

By presenting the method in the main text, we aim to offer a more intuitive understanding of the architectural modifications, while retaining the full technical details in the *Methods* section.

Line number: 75-110.

Minor comments

Reviewer Comment 3.7 — The original Geneformer paper showed scaling with pretraining data at various intervals (100k, 1M, etc). It would be useful to see more data points to determine what the curve looks like. The remaining points are smaller data so should be more efficient to train than the ones the authors already trained.

Reply:

Evaluating performance across multiple pretraining data scales is indeed important for understanding scaling behavior, as highlighted both by the reviewer and prior work [7].

To address this, we extended our experiments to include additional data scale intervals. Specifically, we pretrained both GF and GF_{CAB} on subsets of increasing size (10K, 20K, 100K, 1M, and 30M cells). Following pretraining, we evaluated masked token prediction performance as well as downstream classification performance on the tissue type dataset. The results are summarized in Fig 10.

We observe a consistent improvement in both pretraining and downstream performance as data scale increases for both model architectures. However, the majority of performance gains occur at smaller data scales (up to approximately 1M cells), with diminishing returns observed beyond this point. Increasing the dataset size from 1M to 30M yields comparatively modest improvements, suggesting that much of the learnable signal is captured early in the scaling regime.

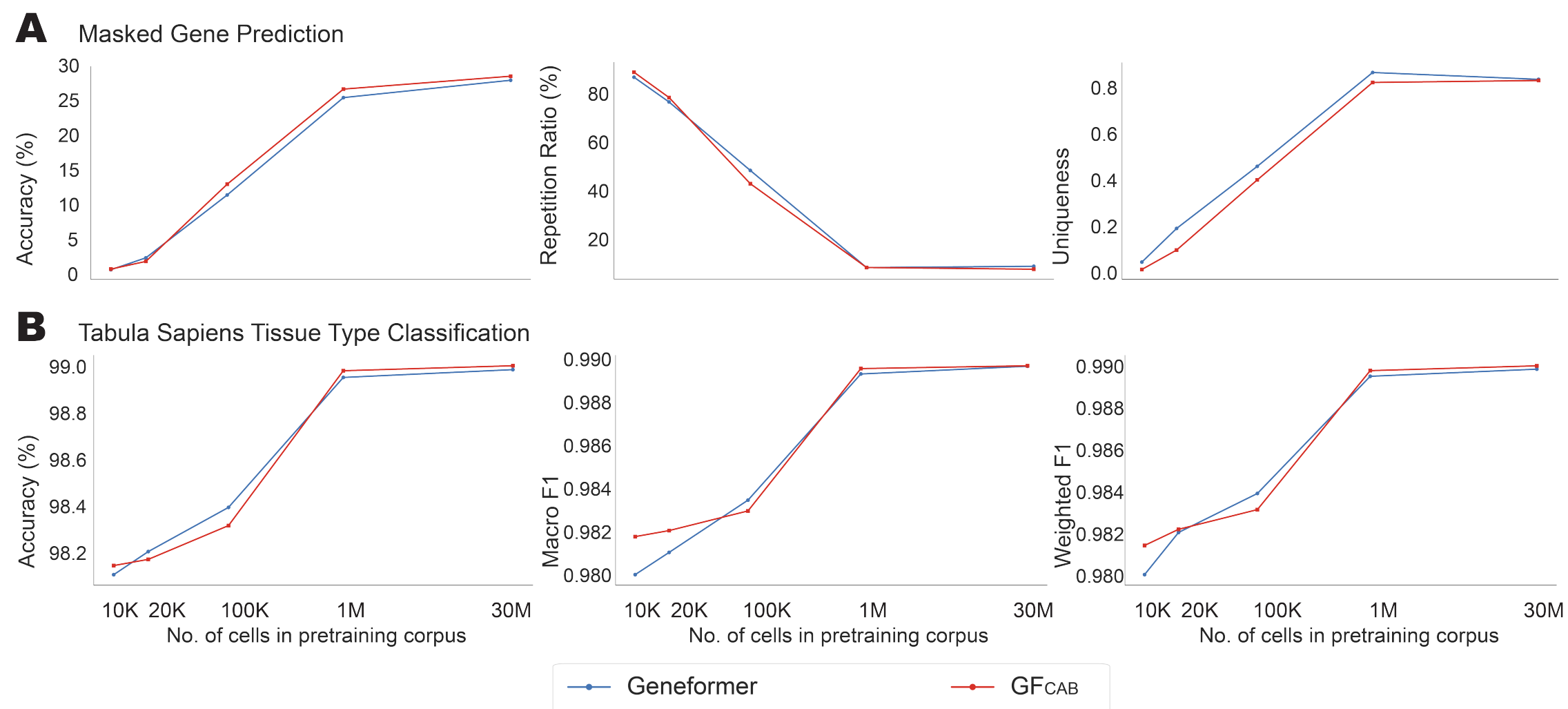

Figure 10: Scaling behavior of GF and GF_{CAB} across different pretraining data sizes (10K, 100K, 1M, and 30M cells). (A) Pretraining performance, including masked token prediction accuracy, repetition ratio, and uniqueness. (B) Downstream tissue-type classification performance, measured by accuracy, macro F1, and weighted F1 scores. Both models show performance improvements with increasing data scale, with diminishing returns observed beyond 1M cells.

Across all data scales, GF and GF_{CAB} exhibit similar scaling trends in both pretraining and downstream evaluations. Notably, GF_{CAB} maintains its advantages in reducing repetition, while achieving comparable or slightly improved performance relative to GF across different scales.

These results provide a more complete characterization of data scaling behavior and support the conclusion that moderate data scales, when combined with appropriate architectural modifications, can achieve strong performance with improved efficiency.

Line number: 145-147.

Reviewer Comment 3.8 — When the authors say cardiomyocyte cell type classification, do they mean disease classification (non-failing, hypertrophic, dilated) or cell type (atrial vs. ventricular cardiomyocytes or other subtypes in the original dataset)?

Reply:

The reviewer is correct to point out this ambiguity. In this study, the term "cardiomyocyte cell type classification" refers to disease state classification, specifically distinguishing between non-failing, hypertrophic, and dilated cardiomyopathy conditions, rather than classification of cardiomyocyte subtypes (*e.g.*, atrial *vs.* ventricular).

We have revised the manuscript to explicitly clarify this terminology and avoid potential confusion.

Line number: 203-213.

References

[1] Kedzierska KZ, Crawford L, Amini AP, Lu AX. Assessing the limits of zero-shot foundation models in single-cell biology; 2023. Available from: http://biorxiv.org/lookup/doi/10.1101/2023.10.16.561085. doi:10.1101/2023.10.16.561085.

[2] Kedzierska KZ, Crawford L, Amini AP, Lu AX. Zero-shot evaluation reveals limitations of single-cell foundation models. Genome Biology. 2025 Apr;26(1):101. Available from: https://genomebiology.biomedcentral.com/articles/10.1186/s13059-025-03574-x. doi:10.1186/s13059-025-03574-x.

[3] Yuan X, Zhan Z, Zhang Z, Zhou M, Zhao J, Han B, et al.. Cell-ontology guided transcriptome foundation model. arXiv; 2024. Version Number: 2. Available from: https://arxiv.org/abs/2408.12373. doi:10.48550/ARXIV.2408.12373.

[4] Cui H, Wang C, Maan H, Pang K, Luo F, Duan N, et al. scGPT: toward building a foundation model for single-cell multi-omics using generative AI. Nature Methods. 2024 Aug;21(8):1470-80. Available from: https://www.nature.com/articles/s41592-024-02201-0. doi:10.1038/s41592-024-02201-0.

[5] Gayoso A, Lopez R, Xing G, Boyeau P, Valiollah Pour Amiri V, Hong J, et al. A Python library for probabilistic analysis of single-cell omics data. Nature Biotechnology. 2022 Feb;40(2):163-6. Available from: https://www.nature.com/articles/s41587-021-01206-w. doi:10.1038/s41587-021-01206-w.

[6] Selewa A, Dohn R, Eckart H, Lozano S, Xie B, Gauchat E, et al. Systematic Comparison of High-throughput Single-Cell and Single-Nucleus Transcriptomes during Cardiomyocyte Differentiation. Scientific Reports. 2020 Jan;10(1):1535. Available from: https://www.nature.com/articles/s41598-020-58327-6. doi:10.1038/s41598-020-58327-6.

[7] Theodoris CV, Xiao L, Chopra A, Chaffin MD, Al Sayed ZR, Hill MC, et al. Transfer learning enables predictions in network biology. Nature. 2023 Jun;618(7965):616-24. Available from: https://www.nature.com/articles/s41586-023-06139-9. doi:10.1038/s41586-023-06139-9.

[8] Tang F, Barbacioru C, Wang Y, Nordman E, Lee C, Xu N, et al. mRNA-Seq whole-transcriptome analysis of a single cell. Nature Methods. 2009 May;6(5):377-82. Available from: https://www.nature.com/articles/nmeth.1315. doi:10.1038/nmeth.1315.

[9] Navin N, Kendall J, Troge J, Andrews P, Rodgers L, McIndoo J, et al. Tumour evolution inferred by single-cell sequencing. Nature. 2011 Apr;472(7341):90-4. Available from: https://www.nature.com/articles/nature09807. doi:10.1038/nature09807.

[10] Bommasani R, Hudson DA, Adeli E, Altman R, Arora S, von Arx S, et al.. On the Opportunities and Risks of Foundation Models. arXiv; 2021. Version Number: 3. Available from: https://arxiv.org/abs/2108.07258. doi:10.48550/ARXIV.2108.07258.

[11] Ramesh A, Pavlov M, Goh G, Gray S, Voss C, Radford A, et al.. Zero-Shot Text-to-Image Generation. arXiv; 2021. Version Number: 2. Available from: https://arxiv.org/abs/2102.12092. doi:10.48550/ARXIV.2102.12092.

[12] Wu Y, Wershof E, Schmon SM, Nassar M, Osiński B, Eksi R, et al.. PerturBench: Benchmarking Machine Learning Models for Cellular Perturbation Analysis. arXiv; 2024. Version Number: 4. Available from: https://arxiv.org/abs/2408.10609. doi:10.48550/ARXIV.2408.10609.

[13] Wang Y, Liu X, Fan Y, Xie B, Cheng J, Wong KC, et al. Predicting drug responses of unseen cell types through transfer learning with foundation models. Nature Computational Science. 2025 Oct;6(1):39-52. Available from: https://www.nature.com/articles/s43588-025-00887-6. doi:10.1038/s43588-025-00887-6.

[14] Theodoris C, Li M, White M, Liu L, He D, Pollard K, et al. Human Disease Modeling Reveals Integrated Transcriptional and Epigenetic Mechanisms of NOTCH1 Haploinsufficiency. Cell. 2015 Mar;160(6):1072-86. Available from: https://linkinghub.elsevier.com/retrieve/pii/S0092867415002019. doi:10.1016/j.cell.2015.02.035.

[15] Jin Y, He Y, Liu B, Zhang X, Song C, Wu Y, et al. Single-cell RNA sequencing reveals the dynamics and heterogeneity of lymph node immune cells during acute and chronic viral infections. Frontiers in Immunology. 2024 Jan;15:1341985. Available from: https://www.frontiersin.org/articles/10.3389/fimmu.2024.1341985/full. doi:10.3389/fimmu.2024.1341985.

[16] Soneson C, Robinson MD. Bias, robustness and scalability in single-cell differential expression analysis. Nature Methods. 2018 Apr;15(4):255-61. Available from: https://www.nature.com/articles/nmeth.4612. doi:10.1038/nmeth.4612.

[17] Xu C, Lopez R, Mehlman E, Regier J, Jordan MI, Yosef N. Probabilistic harmonization and annotation of single-cell transcriptomics data with deep generative models. Molecular Systems Biology. 2021 Jan;17(1):e9620. Available from: https://www.embopress.org/doi/10.15252/msb.20209620. doi:10.15252/msb.20209620.

[18] Roohani Y, Huang K, Leskovec J. Predicting transcriptional outcomes of novel multigene perturbations with GEARS. Nature Biotechnology. 2024 Jun;42(6):927-35. Available from: https://www.nature.com/articles/s41587-023-01905-6. doi:10.1038/s41587-023-01905-6.

[19] Vaswani A, Shazeer N, Parmar N, Uszkoreit J, Jones L, Gomez AN, et al.. Attention Is All You Need. arXiv; 2017. Version Number: 7. Available from: https://arxiv.org/abs/1706.03762. doi:10.48550/ARXIV.1706.03762.

[20] Hao M, Gong J, Zeng X, Liu C, Guo Y, Cheng X, et al. Large-scale foundation model on single-cell transcriptomics. Nature Methods. 2024 Aug;21(8):1481-91. Available from: https://www.nature.com/articles/s41592-024-02305-7. doi:10.1038/s41592-024-02305-7.

[21] Heimberg G, Kuo T, DePianto D, Heigl T, Diamant N, Salem O, et al.. Scalable querying of human cell atlases via a foundational model reveals commonalities across fibrosis-associated macrophages; 2023. Available from: http://biorxiv.org/lookup/doi/10.1101/2023.07.18.549537. doi:10.1101/2023.07.18.549537.

[22] Brown TB, Mann B, Ryder N, Subbiah M, Kaplan J, Dhariwal P, et al.. Language Models are Few-Shot Learners. arXiv; 2020. Version Number: 4. Available from: https://arxiv.org/abs/2005.14165. doi:10.48550/ARXIV.2005.14165.

[23] Yang F, Wang W, Wang F, Fang Y, Tang D, Huang J, et al. scBERT as a large-scale pretrained deep language model for cell type annotation of single-cell RNA-seq data. Nature Machine Intelligence. 2022 Sep;4(10):852-66. Available from: https://www.nature.com/articles/s42256-022-00534-z. doi:10.1038/s42256-022-00534-z.

[24] Wang H, Leskovec J, Regev A. Limitations of cell embedding metrics assessed using drifting islands. Nature Biotechnology. 2025 Jun. Available from: https://www.nature.com/articles/s41587-025-02702-z. doi:10.1038/s41587-025-02702-z.

[25] Pisco A. Tabula Sapiens v2. figshare; 2024. Artwork Size: 57136733314 Bytes Pages: 57136733314 Bytes. Available from: https://figshare.com/articles/dataset/Tabula_Sapiens_v2/27921984. doi:10.6084/M9.FIGSHARE.27921984.

[26] Adams TS, Schupp JC, Poli S, Ayaub EA, Neumark N, Ahangari F, et al. Single-cell RNA-seq reveals ectopic and aberrant lung-resident cell populations in idiopathic pulmonary fibrosis. Science Advances. 2020 Jul;6(28):eaba1983. Available from: https://www.science.org/doi/10.1126/sciadv.aba1983. doi:10.1126/sciadv.aba1983.

[27] Luecken MD, Büttner M, Chaichoompu K, Danese A, Interlandi M, Mueller MF, et al. Benchmarking atlas-level data integration in single-cell genomics. Nature Methods. 2022 Jan;19(1):41-50. Available from: https://www.nature.com/articles/s41592-021-01336-8. doi:10.1038/s41592-021-01336-8.

[28] Zheng GXY, Terry JM, Belgrader P, Ryvkin P, Bent ZW, Wilson R, et al. Massively parallel digital transcriptional profiling of single cells. Nature Communications. 2017 Jan;8(1):14049. Available from: https://www.nature.com/articles/ncomms14049. doi:10.1038/ncomms14049.

[29] Domínguez Conde C, Xu C, Jarvis LB, Rainbow DB, Wells SB, Gomes T, et al. Cross-tissue immune cell analysis reveals tissue-specific features in humans. Science. 2022 May;376(6594):eabl5197. Available from: https://www.science.org/doi/10.1126/science.abl5197. doi:10.1126/science.abl5197.
