# Supplementary material for: Assessing scale and predictive diversity in models for single-cell transcriptomics based on Geneformer
Source: PLoS Comput Biol. 2026 Jul 30;22(7):e1013701. doi: 10.1371/journal.pcbi.1013701 (PMC13456263; doi:10.1371/journal.pcbi.1013701)
Supplement: Peer review history

# Peer review: Decision Letter 0

**DOI:** 10.1371/journal.pcbi.1013701.r001

**Authors:** Michael Domaratzki, Jean Fan

13 Jan 2026

PCOMPBIOL-D-25-02280

Assessing Scale and Predictive Diversity in Models for Single-Cell Transcriptomics based on Geneformer

PLOS Computational Biology

Dear Dr. Schmidt,

Thank you for submitting your manuscript to PLOS Computational Biology. After careful consideration, we feel that it has merit but does not fully meet PLOS Computational Biology's publication criteria as it currently stands. Therefore, we invite you to submit a revised version of the manuscript that addresses the points raised during the review process.

Please submit your revised manuscript by Mar 15 2026 11:59PM. If you will need more time than this to complete your revisions, please reply to this message or contact the journal office at ploscompbiol@plos.org. When you're ready to submit your revision, log on to https://www.editorialmanager.com/pcompbiol/ and select the 'Submissions Needing Revision' folder to locate your manuscript file.

Please include the following items when submitting your revised manuscript:

* A letter that responds to each point raised by the editor and reviewer(s). You should upload this letter as a separate file labeled 'Response to Reviewers'. This file does not need to include responses to formatting updates and technical items listed in the 'Journal Requirements' section below.

* A marked-up copy of your manuscript that highlights changes made to the original version. You should upload this as a separate file labeled 'Revised Manuscript with Track Changes'.

* An unmarked version of your revised paper without tracked changes. You should upload this as a separate file labeled 'Manuscript'.

If you would like to make changes to your financial disclosure, competing interests statement, or data availability statement, please make these updates within the submission form at the time of resubmission. Guidelines for resubmitting your figure files are available below the reviewer comments at the end of this letter

We look forward to receiving your revised manuscript.

Kind regards,

Michael Domaratzki

Academic Editor

PLOS Computational Biology

Jean Fan

Section Editor

PLOS Computational Biology

**Journal Requirements:**

1) We ask that a manuscript source file is provided at Revision. Please upload your manuscript file as a .doc, .docx, .rtf or .tex. If you are providing a .tex file, please upload it under the item type u2018LaTeX Source Fileu2019 and leave your .pdf version as the item type u2018Manuscriptu2019.

2) Please upload all main figures as separate Figure files in .tif or .eps format. For more information about how to convert and format your figure files please see our guidelines:

https://journals.plos.org/ploscompbiol/s/figures

3) Please amend your detailed Financial Disclosure statement. This is published with the article. It must therefore be completed in full sentences and contain the exact wording you wish to be published.

State the initials, alongside each funding source, of each author to receive each grant. For example: "This work was supported by the National Institutes of Health (####### to AM; ###### to CJ) and the National Science Foundation (###### to AM)."

State what role the funders took in the study. If the funders had no role in your study, please state: "The funders had no role in study design, data collection and analysis, decision to publish, or preparation of the manuscript.".

If you did not receive any funding for this study, please simply state: u201cThe authors received no specific funding for this work.u201d

**Reviewers' comments:**

Reviewer's Responses to Questions

**Comments to the Authors:**

**Please note here if the review is uploaded as an attachment.**

Reviewer #1: Summary

The manuscript evaluates how pre-training scale and architectural adjustments influence the performance of Geneformer-based foundation models for single-cell transcriptomics. The authors introduce the GF-CAB variant and benchmark both Geneformer and GF-CAB across masked gene prediction, several classification tasks, and zero-shot batch-effect mitigation. The work addresses a relevant topic: the growing assumption that models trained on larger datasets will improve downstream performance.

Major comments

The cumulative-assignment and similarity-based regularization components are reasonable attempts to address a known issue: repetition of the same gene across multiple masked positions. The manuscript would benefit from a clearer comparison to simpler alternatives. For example, would re-assigning a fixed "first rank position" to each gene and enforcing unique predictions across positions achieve similar redundancy reduction?

The paper places substantial emphasis on repetition and uniqueness as metrics of predictive diversity. However, the biological relevance of these changes is not explored. It would improve the manuscript if the authors examined which additional genes are recovered by GF-CAB (or by smaller-scale models) and whether these genes include rare markers, tissue-specific genes, or otherwise informative features.

The observation that larger-scale pretraining leads to worse zero-shot batch integration is interesting and deserves more discussion. While I understand that diagnosing the precise mechanism may be outside the scope of this study, including a short, dedicated discussion section on possible explanations would help contextualize this result.

Both of the proposed modifications: cumulative-assignment suppression and similarity-based regularization; directly encourage the model to avoid repeated predictions across masked positions. Because repetition and uniqueness are also part of the evaluation metrics, it is difficult to determine how much of the reported improvement reflects better modeling versus optimization toward the metric itself. A discussion of this potential circularity, or an evaluation using alternative diversity measures, would make the claims stronger.

Minor comments

Figure 1.3 (Fig. 3 in the manuscript) is difficult to parse. The large number of tasks, models, and ranking metrics results in very dense panels. A redesign that separates task families or uses clearer labeling would help readers interpret the results more easily.

The introduction mixes broad method families (e.g., MLPs, DET) with specific models (e.g., GEARS, scANVI). Reorganizing this section to separate (i) general methodological families and (ii) representative examples would improve clarity.

Conclusion

The manuscript supports its central conclusion that scale alone does not guarantee better downstream performance, within the scope of the presented evaluations. This aligns with emerging findings from other recent assessments of single-cell foundation models. Additional biological interpretation and deeper contextual discussion would further strengthen the contribution.

Reviewer #2: This paper evaluates whether scaling pre-training data improves the performance of rank-based single-cell foundation models and introduces GFCAB, a modified Geneformer architecture aimed at increasing prediction diversity. Models trained on 1M versus 30M ranked single-cell profiles are assessed on masked-gene prediction, three classification tasks, and zero-shot batch-effect integration. Results show that larger datasets improve masked-token accuracy but do not consistently enhance downstream performance; smaller models sometimes generalize better. Rank-based approaches remain weak for batch correction compared to domain-specific tools. GFCAB improves predictive diversity and reduces reliance on large pre-training corpora but still trails specialized methods.

Major Comments

1. Choice of baseline model is scientifically questionable

Geneformer (GF) is already well documented to underperform compared to more recent single-cell foundation models such as scGPT, scFoundation, and SCimilarity (Kedzierska et al., Genome Biology 2025). These methods incorporate numerical expression values rather than only rank-order information. The study’s rationale is therefore weak unless the authors also include these stronger baselines. Evaluating GF and GF-CAB against these non-rank-based models under the same evaluation framework would provide essential context and greatly strengthen the study.

2. Incorrect claim regarding the first transformer-based single-cell model

The manuscript states that GF was the first BERT-style model for single-cell transcriptomics. This is factually incorrect. scBERT preceded Geneformer and should be acknowledged appropriately.

3. Missing comparison to scCello, a key rank-based foundation model

Another rank-based model, scCello, uses cell ontology to guide pre-training. The authors use scCello’s cell-type data but do not compare against the scCello model itself. This comparison is essential to determine whether GFCAB offers improvements over other augmented rank-based methods beyond GF alone.

4. Lack of explanation for greater diversity in smaller-scale models

The manuscript claims that smaller-scale models exhibit greater exploratory diversity, but offers no mechanistic or empirical explanation. This is a central observation and should be justified biologically, algorithmically, or statistically.

5. Missing specification of “Other Models” in Fig. 3A

Figure 3A reports rankings among “Other Models,” but the manuscript does not specify what those models are. For meaningful interpretation, the figure should explicitly list scVI, Harmony, or other clustering/integration tools, as well as supervised baselines (e.g., SVM, logistic regression).

6. The fact that GFCAB-30M underperforms GF-30M needs more explanation

The observation that GFCAB-30M performs worse than GF-30M in certain tasks (Fig. 3B–C) contradicts the stated motivation for model refinements. The manuscript must explain this behavior—whether due to over-regularization, suboptimal hyperparameters, or model instability.

7. Study examines data scaling but not model scaling

The authors explore the effect of data scale (1M vs. 30M profiles) but not model scale. Scaling laws in transformers often require increased parameter counts—e.g., deeper networks, larger embedding dimensions—for benefits from larger datasets to manifest. The authors should discuss (or ideally test) whether GF or GFCAB benefits more from increased model capacity at larger data scales.

Minor Comments

1. Figure 1 font size too small

The text and labels in Fig. 1 are difficult to read and should be enlarged.

2. Present numerical values instead of rankings in Fig. 3A

It would be more informative to include raw numerical performance metrics rather than a ranking-only figure. Best-performing methods should be bolded. Typically, this information is conveyed via a table rather than a figure.

Reviewer #3: The authors propose an improvement to Geneformer to limit repetition of genes and similarity of predictions for masked learning and test the performance of this method vs. the original Geneformer with 10 or 30M cell pretraining datasets on several downstream tasks, also comparing to baselines. The authors test both biologically-relevant downstream tasks (gene dosage sensitivity, cardiomyocyte cell type classification, and tissue-specific cell type classification) as well as the preprocessing task of batch correction.

The study is a useful comparison of different dataset sizes and an interesting architectural modification to improve the pretraining. However, the overarching conclusions of the paper as written seem to focus almost entirely on the batch effect results and ignore the biologically-relevant task results. At the end of the day, the biologically relevant tasks are what biologists would like to study, and the batch effect correction is simply a preprocessing means to an end. One finding in the data of the paper that is not discussed by the authors is that it appears that batch effect integration is correlated with worse performance on biologically relevant tasks. This raises the question of why the field is so focused on batch effect integration if it actually leads to worse performance on the tasks relevant to the true biological questions at hand. Overall, the text should be revised to not cherry pick the results to overweight the batch effect correction, and should instead objectively report the findings.

Major points:

- Fig. 3 indicates that the Geneformer variants significantly outperformed other models in all of the biologically relevant tasks. If there are lower batch effects but the models do worse in distinguishing biologically relevant features, then it seems that batch effect integration does not need to be optimized for these tasks and in fact correcting the batch effects may be removing true biological signal that is necessary for the biologically relevant tasks. It is unclear why the authors would equally weight the 1 task of batch correction on 3 datasets with the 3 distinct biological tasks (which are folded into 1 for the averaging). Since batch effects are a preprocessing task for the purpose of improving biological task performance, it would seem the biological tasks should take priority, rather than reducing them to a single point. This cherry-picking of the results to overweight batch correction in the discussion leads to the conclusions of the paper not being well-supported by the results.

- Scaling is not only about increasing data but also increasing model size accordingly. There is generally a number of parameters that are ideal for a given amount of data, and so adding more data without increasing the size of the model would have no benefit, whereas increasing the size of the model would indeed show a benefit. The authors should test this and should also report the parameter size of the model, as this was not readily noted in the text.

- Since the dataset 1M is simply a subsample of 30M and represents the same diversity, the paper seems to analyze the net benefit of training with the rest of the 29M cells. However, the 1M subsample benefits from the diversity of the data curation efforts for 30M cells, so is not really indicative of training with only a 1M cell corpus. The authors should revise the text to make this clear. If one imagines a loss curve, most of the gains are had in the beginning portion on the compute optimal frontier, so the authors are likely essentially testing the difference between stopping the training early or continuing for the remainder of the 30M cells.

- The authors should add baselines to the pretraining accuracy such as the naive method of selecting the most common gene for that position. For example, if Gene A was the most commonly located within the pretraining corpus at position 3, then the naive guess for position 3 would be Gene A. (The top likely gene should be calculated from the pretraining corpus and not the held out data on which the prediction is being performed.)

- The original paper indicated that batch effects are improved by fine-tuning on biologically relevant labels. Since other tools are explicitly designed for batch correction, the authors should test Geneformer with the suggested batch effect correction method. For example, the original paper performed cell type fine-tuning on single-cell RNAseq data and tested the batch integration of single-nucleus RNAseq data without further training.

- Currently the architectural modifications proposed by the authors are not sufficiently discussed in the main text of the paper. The method should be further outlined in the main text so readers have an intuitive understanding of the modifications.

Minor points:

- The original Geneformer paper showed scaling with pretraining data at various intervals (100k, 1M, etc). It would be useful to see more data points to determine what the curve looks like. The remaining points are smaller data so should be more efficient to train than the ones the authors already trained.

- When the authors say cardiomyocyte cell type classification, do they mean disease classification (non-failing, hypertrophic, dilated) or cell type (atrial vs. ventricular cardiomyocytes or other subtypes in the original dataset)?

**********

**Have the authors made all data and (if applicable) computational code underlying the findings in their manuscript fully available?**

The PLOS Data policy requires authors to make all data and code underlying the findings described in their manuscript fully available without restriction, with rare exception (please refer to the Data Availability Statement in the manuscript PDF file). The data and code should be provided as part of the manuscript or its supporting information, or deposited to a public repository. For example, in addition to summary statistics, the data points behind means, medians and variance measures should be available. If there are restrictions on publicly sharing data or code —e.g. participant privacy or use of data from a third party—those must be specified.

Reviewer #1: Yes

Reviewer #2: Yes

Reviewer #3: Yes

**********

PLOS authors have the option to publish the peer review history of their article (what does this mean?). If published, this will include your full peer review and any attached files.

If you choose “no”, your identity will remain anonymous but your review may still be made public.

**Do you want your identity to be public for this peer review?**  For information about this choice, including consent withdrawal, please see our Privacy Policy.

Reviewer #1: No

Reviewer #2: No

Reviewer #3: No

[NOTE: If reviewer comments were submitted as an attachment file, they will be attached to this email and accessible via the submission site. Please log into your account, locate the manuscript record, and check for the action link "View Attachments". If this link does not appear, there are no attachment files.]

**Figure resubmission:**

While revising your submission, we strongly recommend that you use PLOS’s NAAS tool (https://ngplosjournals.pagemajik.ai/artanalysis) to test your figure files. NAAS can convert your figure files to the TIFF file type and meet basic requirements (such as print size, resolution), or provide you with a report on issues that do not meet our requirements and that NAAS cannot fix.

After uploading your figures to PLOS’s NAAS tool - https://ngplosjournals.pagemajik.ai/artanalysis, NAAS will process the files provided and display the results in the "Uploaded Files" section of the page as the processing is complete. If the uploaded figures meet our requirements (or NAAS is able to fix the files to meet our requirements), the figure will be marked as "fixed" above. If NAAS is unable to fix the files, a red "failed" label will appear above. When NAAS has confirmed that the figure files meet our requirements, please download the file via the download option, and include these NAAS processed figure files when submitting your revised manuscript.

**Reproducibility:**

To enhance the reproducibility of your results, we recommend that authors of applicable studies deposit laboratory protocols in protocols.io, where a protocol can be assigned its own identifier (DOI) such that it can be cited independently in the future. Additionally, PLOS ONE offers an option to publish peer-reviewed clinical study protocols. Read more information on sharing protocols at https://plos.org/protocols?utm_medium=editorial-email&utm_source=authorletters&utm_campaign=protocols

# Author comment: Author response to Decision Letter 1

**DOI:** 10.1371/journal.pcbi.1013701.r002

12 May 2026

Attachment Submitted filename: Response_Letter.pdf

# Peer review: Decision Letter 1

**DOI:** 10.1371/journal.pcbi.1013701.r003

**Authors:** Michael Domaratzki, Jean Fan

2 Jul 2026

Dear Dr Schmidt,

We are pleased to inform you that your manuscript 'Assessing scale and predictive diversity in models for single-cell transcriptomics based on Geneformer' has been provisionally accepted for publication in PLOS Computational Biology.

Before your manuscript can be formally accepted you will need to complete some formatting changes, which you will receive in a follow up email. A member of our team will be in touch with a set of requests.

Please note that your manuscript will not be scheduled for publication until you have made the required changes, so a swift response is appreciated.

IMPORTANT: The editorial review process is now complete. PLOS will only permit corrections to spelling, formatting or significant scientific errors from this point onwards. Requests for major changes, or any which affect the scientific understanding of your work, will cause delays to the publication date of your manuscript.

Should you, your institution's press office or the journal office choose to press release your paper, you will automatically be opted out of early publication. We ask that you notify us now if you or your institution is planning to press release the article. All press must be co-ordinated with PLOS.

Thank you again for supporting Open Access publishing; we are looking forward to publishing your work in PLOS Computational Biology.

Best regards,

Jean Fan

Section Editor

PLOS Computational Biology

Jean Fan

Section Editor

PLOS Computational Biology

***********************************************************

Overall, the authors have added analyses to address reviewer concerns. The authors should address the minor outstanding reviewer concerns regarding language to refrain from suggesting unsubstantiated benefits of scBERT and speculating on reasons behind improved in-distribution performance for Geneformer. But at this stage, re-evaluation by reviewers is no longer needed and we are happy to accept the manuscript in principle.

Reviewer's Responses to Questions

**Comments to the Authors:**

**Please note here if the review is uploaded as an attachment.**

Reviewer #2: All my comments were addressed adequately. Thanks authors for the efforts.

Reviewer #3: Overall, the authors have added analyses to address the reviewer points. There are a few points remaining to address based on changes made in the last review.

- The authors correctly added to the introduction that scBERT applied the BERT architecture to single-cell settings. However, the scBERT paper did not "demonstrate strong performance across a wide range of downstream tasks" as the manuscript only applied scBERT for cell type classification, while Geneformer was the first to demonstrate the BERT architecture as a foundation model pretrained on single-cell RNAseq data that can be applied to a wide range of downstream tasks. The authors should clarify this in the introduction. (Lines 18-23)

- In the original Geneformer paper, the authors tested the dosage sensitivity task comparing the performance when the cells were included in the pretraining corpus vs. not, and found no difference in the performance. As such, it is unlikely that memorization is the reason GF-30M outperforms in this task. The authors should thus revise the statement that "In such settings, GF-30M benefits from stronger memorization of dataset-specific structures, leading to improved in-distribution performance" or repeat the pretraining with a held out sample of cells to perform this task and provide support for their statement. (Lines 201-202)

- In Fig. 8, where the authors add the baseline result, the line is dashed whereas the legend has a solid line, which may lead to confusion. The authors should modify the legend to match the line on the plot. It also may be easier to see with a brighter color, especially given the baseline is very low in panel B.

**********

**Have the authors made all data and (if applicable) computational code underlying the findings in their manuscript fully available?**

The PLOS Data policy requires authors to make all data and code underlying the findings described in their manuscript fully available without restriction, with rare exception (please refer to the Data Availability Statement in the manuscript PDF file). The data and code should be provided as part of the manuscript or its supporting information, or deposited to a public repository. For example, in addition to summary statistics, the data points behind means, medians and variance measures should be available. If there are restrictions on publicly sharing data or code —e.g. participant privacy or use of data from a third party—those must be specified.

Reviewer #2: Yes

Reviewer #3: None

**********

PLOS authors have the option to publish the peer review history of their article (what does this mean?). If published, this will include your full peer review and any attached files.

If you choose “no”, your identity will remain anonymous but your review may still be made public.

**Do you want your identity to be public for this peer review?**  For information about this choice, including consent withdrawal, please see our Privacy Policy.

Reviewer #2: No

Reviewer #3: No

# Editor report: Acceptance letter

**DOI:** 10.1371/journal.pcbi.1013701.r004

**Authors:** Michael Domaratzki, Jean Fan

PCOMPBIOL-D-25-02280R1

Assessing scale and predictive diversity in models for single-cell transcriptomics based on Geneformer

Dear Dr Schmidt,

I am pleased to inform you that your manuscript has been formally accepted for publication in PLOS Computational Biology. Your manuscript is now with our production department and you will be notified of the publication date in due course.

The corresponding author will soon be receiving a typeset proof for review, to ensure errors have not been introduced during production. Please review the PDF proof of your manuscript carefully, as this is the last chance to correct any errors. Please note that major changes, or those which affect the scientific understanding of the work, will likely cause delays to the publication date of your manuscript.

Soon after your final files are uploaded, unless you have opted out, the early version of your manuscript will be published online. The date of the early version will be your article's publication date. The final article will be published to the same URL, and all versions of the paper will be accessible to readers.

For Research, Software, and Methods articles, you will receive an invoice from PLOS for your publication fee after your manuscript has reached the completed accept phase. If you receive an email requesting payment before acceptance or for any other service, this may be a phishing scheme. Learn how to identify phishing emails and protect your accounts at https://explore.plos.org/phishing.

Thank you again for supporting PLOS Computational Biology and open-access publishing. We are looking forward to publishing your work!

With kind regards,

Janani Seenivasan

PLOS Computational Biology | Carlyle House, Carlyle Road, Cambridge CB4 3DN | United Kingdom ploscompbiol@plos.org | Phone +44 (0) 1223-442824 | ploscompbiol.org | @PLOSCompBiol
